# Human brain connectome profiles mediate the relationship between pathology burden and clinical phenotypes in Alzheimer's disease

**DOI:** 10.1002/alz.71638

**Published:** 2026-06-30

**Authors:** Yating Li, Dong Wang, Rongshen Zhou, Shaozhen Yan, Dawei Wang, Yongbin Wei, Hongxiang Yao, Bo Zhou, Jie Lu, Pan Wang, Zhengluan Liao, Ying Han, Xi Zhang, Yihe Zhang, Yong Liu, Kun Zhao, for the Alzheimer's Disease Neuroimaging Initiative

**Affiliations:** ^1^ School of Artificial Intelligence Beijing University of Posts and Telecommunications Beijing China; ^2^ Queen Mary School Hainan Beijing University of Posts and Telecommunications Lingshui Hainan China; ^3^ Department of Radiology Xuanwu Hospital of Capital Medical University Beijing China; ^4^ Department of Radiology Qilu Hospital of Shandong University Jinan Shandong China; ^5^ Department of Epidemiology and Health Statistics School of Public Health Shandong University Jinan Shandong China; ^6^ Institute of Brain and Brain‐Inspired Science Shandong University Jinan Shandong China; ^7^ Department of Radiology National Clinical Research Centre for Geriatric Diseases the Second Medical Centre Chinese PLA General Hospital Beijing China; ^8^ Department of Neurology National Clinical Research Centre for Geriatric Diseases the Second Medical Centre Chinese PLA General Hospital Beijing China; ^9^ Department of Neurology Tianjin Huanhu Hospital Tianjin China; ^10^ Department of Psychiatry People's Hospital of Hangzhou Medical College Zhejiang Provincial People's Hospital Hangzhou Zhejiang China; ^11^ Department of Neurology Xuanwu Hospital of Capital Medical University Beijing China; ^12^ National Clinical Research Center for Geriatric Disorders Beijing China; ^13^ Center of Alzheimer's Disease Beijing Institute for Brain Disorders Beijing China

**Keywords:** Alzheimer's disease, brain resilience, clinical heterogeneity, mediator, multimodal brain network

## Abstract

**INTRODUCTION:**

Mild cognitive impairment (MCI), a prodromal stage of Alzheimer's disease (AD), shows pronounced clinical heterogeneity poorly explained by pathology burden, representing a gap complicating prognosis. As the brain operates as a complex network for information integration, we hypothesized that connectome architecture mediates the link between AD pathology and clinical expression.

**METHODS:**

We developed a framework integrating structural and functional connectomes from multi‐center cohorts, performing connectome‐based subtyping in MCI, with analyses of upstream pathology, downstream phenotypes, and transcriptomic associations.

**RESULTS:**

This approach identified an “MCI‐compromised” (MCI‐C) subgroup characterized by extensive structural‐functional connectomic disruption and an “MCI‐preserved” (MCI‐P) subgroup with relatively preserved connectome integrity. Despite comparable pathology, MCI‐C demonstrated more severe neurodegeneration, accelerated cognitive decline, and elevated progression risk. Multiscale analyses linked these patterns to transcriptomic profiles of mitochondrial, synaptic, and neuroimmune processes.

**DISCUSSION:**

These findings demonstrate that the connectome acts as a critical mediator, rather than a passive endophenotype, shaping AD clinical expression.

## Highlights

- Structural–functional connectome fusion captures complementary network information.
- Two connectome‐defined mild cognitive impairment (MCI) subgroups are identified.
- MCI subgroups show similar pathology but divergent neurodegeneration and cognitive trajectories.
- Connectome patterns link to transcriptomic signatures of mitochondrial metabolism, synaptic ion homeostasis, and neuroimmune activation.
- Connectome architecture mediates pathology–phenotype relationships in Alzheimer's disease.

## 1 BACKGROUND

Alzheimer's disease (AD) is the most common cause of dementia, characterized by progressive cognitive decline accompanied by widespread neurobiological alterations.^1^, ^2^, ^3^ The amyloid‐β (Aβ) cascade hypothesis conceptualizes AD as a continuous biological process in which Aβ overproduction and tau hyperphosphorylation gradually trigger downstream changes in brain structure and function, ultimately culminating in clinical dementia.^4^, ^5^ While this model is widely accepted and serves as the gold standard for diagnosis and therapeutic intervention in AD, a stark dissociation often exists between molecular pathology and clinical phenotype. Specifically, levels of Aβ or tau burden frequently diverge sharply from the severity of neurodegeneration and cognitive impairment. This dissociation is particularly prominent during the mild cognitive impairment (MCI) stage. As a prodromal phase of AD, MCI represents a critical transitional period when pathological burden is actively accumulating, yet substantial heterogeneity in clinical profiles has already emerged.^6^, ^7^ This inconsistency indicates that the translation of pathology into clinical phenotype is modulated by additional system‐level factors.^8^

The brain is organized into large‐scale, interconnected networks that support cognitive functions. A convergence of evidence indicates that the organization and dynamics of these networks are fundamental to cognitive performance.^9^ AD is increasingly viewed as a progressive connectome disconnection syndrome, in which disruption of large‐scale structural and functional network organization is closely associated with cognitive decline and clinical progression.^10^, ^11^ Moreover, the network architecture has also been implicated in the spatial distribution and propagation of neurodegenerative pathology, with disease‐related processes preferentially spreading along connectivity pathways.^12^, ^13^ Emerging evidence further suggests that large‐scale brain networks exhibit differential vulnerability and resilience to neurodegenerative pathology, potentially influencing the extent to which pathological burden translates into cognitive impairment.^14^ Together, these findings suggest that connectome architecture serves as a critical mediator—not merely a passive endophenotype—shaping how similar levels of AD pathology translate into distinct structural, functional, and cognitive outcomes.

Characterizing the human connectome is central to understanding this complex regulatory mechanism. Most current approaches characterize human connectome organization using a single imaging modality, such as structural connectivity, functional connectivity, or morphological covariance. However, the brain is a complex, multi‐scale system in which complementary modalities capture distinct but interrelated aspects of connectivity.^15^, ^16^, ^17^, ^18^ Single‐modality descriptions may therefore provide an incomplete representation of network architecture and its role in disease. Integrative, multimodal connectome frameworks offer a more comprehensive characterization of brain organization and may better capture biologically meaningful variation relevant to clinical heterogeneity.

**RESEARCH IN CONTEXT**

1. **Systematic review**: Amyloid‐β and tau models do not fully explain the dissociation between pathology and clinical phenotypes in Alzheimer's disease. Although increasing evidence suggests that large‐scale brain networks support cognition and shape neurodegenerative pathology, most studies rely on single modalities, and a multimodal connectome framework to explain pathology–phenotype heterogeneity in mild cognitive impairment (MCI) remains lacking.
2. **Interpretation**: Using a structural–functional fusion connectome framework, we identify two MCI subgroups with distinct connectome profiles despite similar pathology, showing divergent neurodegeneration and cognitive trajectories. These findings indicate that connectome organization mediates pathology–phenotype relationships in Alzheimer's disease.
3. **Future directions**: Future studies should clarify the causal role of connectome organization in mediating pathology–phenotype relationships and evaluate its clinical utility for patient stratification and personalized intervention. Additionally, integrating additional biological modalities may further refine the understanding of heterogeneity in Alzheimer's disease.

**FIGURE 1:**
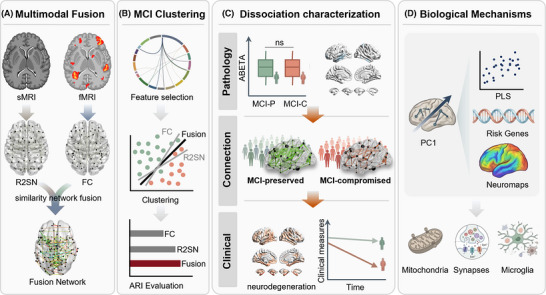
Conceptual framework of the study. (A) The integration of structural and functional brain network to construct a comprehensive multimodal fusion network. (B) Multimodal clustering and evaluation of MCI subgroups. (C) Characterization of the dissociation between pathology and clinical phenotype. (D) Correlation of the principal component of connectome variance with risk genes and multimodal brain maps, followed by GSEA for biological process and cell type identification. Created with BioRender.com. GSEA, gene set enrichment analysis.

In this study, we constructed multimodal brain connectome profiles by integrating complementary imaging‐derived connectivity measures to characterize individual network organization. Using this framework, we demonstrate that distinct connectome profiles are associated with differential coupling between upstream pathology and downstream clinical manifestations. Furthermore, by anchoring these network‐level differences to transcriptomic profiles and neuromaps, we explored the biological processes that may underlie connectome divergence in AD (Figure 1). Collectively, our findings suggest that multimodal network architecture shapes how pathological burden translates into cognitive and clinical outcomes, providing a systems‐level perspective on AD heterogeneity.

## 2 METHODS

### 2.1 Participants

The study included a total of 1152 participants from two independent datasets. The primary discovery dataset was the Multi‐Center Alzheimer Disease Imaging Consortium (MCADI) (n = 786, cognitively normal (CN) = 256, MCI = 251, AD = 279), collected from four hospitals across seven scanner sites in China.^19^, ^20^, ^21^ To test the robustness of our findings and to further characterize the relationships among AD pathology, brain connectome, and clinical phenotype, we additionally included 366 participants (CN = 206, MCI = 123, AD = 37) from the publicly available Alzheimer's Disease Neuroimaging Initiative (ADNI) (http://adni.loni.usc.edu) dataset. All participants had T1‐weighted structural magnetic resonance imaging (MRI), functional MRI (fMRI), and associated clinical data. Specifically, a subset of participants from the ADNI cohort (CN = 29, MCI = 66, AD = 24) had complete molecular biomarker data, including Aβ quantified via cerebrospinal fluid (CSF) and positron emission tomography (PET), as well as CSF measurements of total tau and phosphorylated tau. This subset was utilized for subsequent analyses of upstream pathological processes underlying AD.^22^ See Supporting Information for detailed information.

### 2.2 MRI preprocessing

For T1‐weighted structural images, preprocessing included N4 bias field correction, registration to Montreal Neurological Institute (MNI) space, resampling to 1×1×1 mm^3^ isotropic resolution, and processing via CAT12.^23^ Gray matter volume and cortical thickness were then derived based on the Brainnetome Atlas parcellation. For resting‐state fMRI, initial preprocessing was performed using the fmriprep pipeline, which involved head motion correction, slice timing correction, distortion correction, alignment to structural MRI, and cortical mapping, followed by spatial normalization to MNI space.^24^ Nuisance signal regression was then conducted to remove head motion parameters (plus their first‐order derivatives), as well as signals from cerebrospinal fluid and white matter. Considering that the global signal can artifactually enhance global voxel synchronization and potentially obscure true network‐specific signals, the global mean signal was also regressed out. Subsequently, linear detrending, z‐score standardization, temporal band‐pass filtering (0.01–0.08 Hz), and spatial smoothing (4 mm full‐width half‐maximum [FWHM] kernel) were applied. Finally, regional time series were extracted according to the Brainnetome Atlas.^25^

### 2.3 Whole‐brain functional connectivity and regional radiomics similarity network construction

In this study, two complementary brain networks were analyzed to represent the human connectome. To characterize the structural connectome, we constructed regional radiomics similarity network (R2SN) — a structural covariance model based on radiomic features. For each brain region, 25 radiomic features were extracted from the T1‐weighted images. These features were normalized within each participant to reduce inter‐individual variability. A symmetric R2SN matrix was then generated by calculating Pearson correlations between the radiomic feature vectors of every pair of regions, representing inter‐regional structural similarity. Functional connectivity (FC) networks were constructed by computing Pearson correlation coefficients between the regional mean blood oxygen‐level dependent (BOLD) time series across all region pairs, resulting in a symmetric FC matrix for each participant.

### 2.4 Multimodal network fusion and validation

To integrate the functional and structural networks, we employed a similarity network fusion (SNF) framework.^26^ Briefly, a K‐nearest neighbor graph was first constructed for each modality to encode local similarity. Through an iterative nonlinear diffusion process, the state matrices of each modality were updated, allowing similarity information to propagate within local neighborhoods and be exchanged across modalities. This process reinforced concordant multimodal patterns while reducing modality‐specific noise, ultimately converging to a single, symmetric fusion network on a normalized scale. Based on prior methodological recommendations and systematic parameter sensitivity analyses, the neighborhood size was set to *K* = 30 and the number of diffusion iterations to *T* = 20 (Figure S1). This configuration ensured stable convergence of the fusion process while preserving meaningful sparsity and local topological structure. Furthermore, we performed variance decomposition using generalized additive models (GAMs) within the CN subgroup to quantify structural and functional contributions to the fused network. The fused edge weights were modeled as the dependent variable. By fitting R2SN and FC connectivity first as independent predictors to estimate individual explained variance, and subsequently as joint predictors, we evaluated their combined explanatory power and complementary information.

### 2.5 AD‐related connection alterations detection

Given that many connections are high‐dimensional or redundant, we screened for the subset that was truly relevant to AD by identifying connections with significant group differences between AD and CN. This was accomplished through connection‐wise two‐sample t‐tests between AD and CN groups, after regressing out age and sex as covariates, with the application of Bonferroni correction to establish a conservative statistical threshold for significance.

### 2.6 MCI subgroups and related clinical manifestations exploration

To explore whether brain network architecture regulates the relationship between pathology and clinical phenotype, we subdivided patients with MCI into distinct subgroups using a K‐means clustering framework applied to the AD‐related connections. The optimal number of clusters was determined using the within‐cluster sum of squares (WSS) criterion, based on the elbow method. To evaluate the robustness of the clustering results, we also repeated the subgroup division using non‐negative matrix factorization (NMF). Consistency between the two clustering outcomes was quantified with the adjusted Rand Index (ARI).

We next characterized the specific alteration patterns in upstream pathology and downstream clinical phenotypes across the identified subgroups. Upstream pathology was assessed primarily via measures of Aβ deposition and tau. Downstream effects were evaluated through neuroimaging hallmarks, including gray matter volume, cortical thickness, and fluorodeoxyglucose (FDG) ‐PET. Group differences in these measures were tested using analysis of covariance (ANCOVA) with post‐hoc comparisons, controlling for age and sex. The significance threshold was adjusted for multiple comparisons via Bonferroni correction.^27^

We further characterized clinical measures of cognitive ability and longitudinal trajectories for each subgroup. Group differences in cognitive outcomes were tested using ANCOVA with age and sex as covariates, with false discovery rate (FDR) correction applied. The longitudinal changes in cognitive and pathological measures were analyzed using linear mixed‐effects models (LMEs) to account for within‐subject repeated measurements, with false discovery rate correction applied. Differences in the risk of progression to AD across subgroups were evaluated using Kaplan–Meier survival analysis and log‐rank tests.

### 2.7 Biological basis analysis of network‐mediated phenotype regulation

To elucidate the biological mechanisms by which brain network architecture regulates the relationship between AD pathology and clinical phenotype, we performed an imaging genetics analysis. First, we used principal component analysis (PCA) to reduce the high‐dimensional connectivity alterations to a regional summary. We then associated the first principal component (PC1) with whole‐brain gene expression profiles from the Allen Human Brain Atlas using partial least squares regression (PLSR).^28^, ^29^ To further determine the relationships between AD‐related gene expression and regional changes in the MCI subgroup, 13 AD‐related genes were first identified by searching the disease term “Alzheimer disease” on the AHBA website (https://human.brain‐map.org/microarray/search).^30^ Then, we computed the Pearson correlation between the regional expression of each gene and the regional PC1 map. Moreover, we correlated the map with multiple brain atlas maps from neuromaps.^31^ Finally, genes were ranked according to their PLSR weights, and gene set enrichment analysis (GSEA) was conducted to identify significantly enriched biological pathways, with significance assessed using FDR correction.^32^

### 2.8 Statistical analysis

Group differences were assessed using ANCOVA. Longitudinal trajectories and progression risk were evaluated using LME models and Kaplan–Meier survival analyses with log‐rank tests, respectively. Spatial and transcriptomic associations were analyzed using Pearson correlation and PLSR. Multiple‐comparison correction strategies were selected according to the number of statistical tests and the correlation structure of the analyses. Bonferroni correction was applied to large‐scale and high‐dimensional analyses, including connectome‐wide analyses and regional neuroimaging analyses. FDR correction was applied to cognitive, longitudinal, transcriptomic, and neuromaps‐related analyses.

### 2.9 Robustness and reproducibility analyses

To evaluate the robustness and reproducibility of the findings, several complementary validation analyses were performed. AD‐related connectomic alterations were restricted to connections consistently identified across the MCADI discovery cohort and the ADNI validation cohort. In addition, MCI subgroup clustering was further validated using an alternative clustering framework based on NMF, with clustering consistency quantified using the ARI. Repeated resampling analyses were also performed to evaluate the stability of subgroup assignments.

For subgroup‐related pathological, cognitive, longitudinal, and progression‐risk analyses, robustness was further evaluated using permutation testing and stratified subsampling analyses. Randomized group‐label permutation tests were performed to assess whether the observed subgroup differences exceeded chance‐level expectations. In parallel, repeated stratified subsampling analyses were conducted to evaluate the stability of subgroup‐related effects under sample perturbation.

To account for the potential influence of spatial autocorrelation in imaging‐transcriptomic and neuromaps analyses, spatial autocorrelation‐preserving permutation testing was performed using BrainSMASH with 1000 surrogate maps. Surrogate brain maps preserving the spatial autocorrelation structure of the original imaging phenotype were generated to construct spatially constrained null distributions for statistical inference.

## 3 RESULTS

### 3.1 Demographic and clinical characteristics

In the primary MCADI cohort, no significant differences were observed in age and sex distribution across diagnostic groups (both *p* > 0.05). Global cognitive performance, as assessed using the Mini‐Mental State Examination (MMSE), differed significantly across groups (*p* < 0.001, one‐way ANOVA) (Table 1). In the independent ADNI cohort, age was comparable among the three groups (*p* > 0.05), whereas sex distribution differed significantly across groups (*p* = 0.001). All cognitive measures—including MMSE, Clinical Dementia Rating Sum of Boxes (CDR‐SB), Alzheimer's Disease Assessment Scale–Cognitive Subscales (ADAS11, ADAS13, ADASQ4), and Rey Auditory Verbal Learning Test (RAVLT) subscores—demonstrated significant group differences (all *p* < 0.001) (Table 2).

**TABLE 1:** Detailed information of the participants from the MCADI dataset in this study.

Site (n = 786)	Parameter	CN (n = 256)	MCI (n = 251)	AD (n = 279)	*p*‐value
Site1 (n = 119)	N	41	34	44	
	Sex (M/F)	19/22	13/21	20/24	0.747
	Age	68.61 ± 6.68	69.50 ± 8.82	69.89 ± 8.81	0.764
	MMSE	28.54 ± 1.42	26.62 ± 2.47	17.34 ± 6.48	<0.001
Site2 (n = 66)	N	21	21	24	
	Sex (M/F)	11/10	11/10	6/18	0.096
	Age	68.62 ± 4.73	74.10 ± 8.01	72.75 ± 8.30	0.044
	MMSE	28.90 ± 1.09	26.76 ± 1.73	19.17 ± 4.64	<0.001
Site3 (n = 94)	N	24	33	37	
	Sex (M/F)	9/15	10/23	18/19	0.286
	Age	65.54 ± 6.16	65.39 ± 8.33	67.68 ± 8.32	0.409
	MMSE	28.75 ± 1.15	25.94 ± 2.50	15.84 ± 5.52	<0.001
Site4 (n = 121)	N	42	16	63	
	Sex (M/F)	12/30	8/8	27/36	0.209
	Age	65.45 ± 6.75	66.12 ± 7.40	67.78 ± 7.23	0.244
	MMSE	28.48 ± 1.74	24.81 ± 1.52	18.86 ± 3.44	<0.001
Site5 (n = 195)	N	64	92	39	
	Sex (M/F)	25/39	46/46	14/25	0.222
	Age	66.59 ± 6.33	67.84 ± 10.06	68.77 ± 8.83	0.449
	MMSE	28.11 ± 2.26	24.16 ± 3.65	16.64 ± 6.65	<0.001
Site6 (n = 69)	N	21	18	30	
	Sex (M/F)	7/14	10/8	14/16	0.368
	Age	65.05 ± 8.16	70.17 ± 7.94	65.27 ± 7.83	0.081
	MMSE	28.48 ± 1.36	21.94 ± 5.05	11.03 ± 6.34	<0.001
Site7 (n = 122)	N	43	37	42	
	Sex (M/F)	20/23	13/24	16/26	0.553
	Age	68.28 ± 7.95	69.78 ± 6.94	70.40 ± 8.68	0.449
	MMSE	28.79 ± 1.22	26.27 ± 2.59	15.83 ± 5.77	<0.001

Abbreviations: MCADI, Multi‐Center Alzheimer Disease Imaging Consortium; MMSE, Mini‐Mental State Examination.

**TABLE 2:** Detailed information of the participants from the ADNI dataset in this study.

Parameter	CN(n = 206)	MCI(n = 123)	AD(n = 37)	*p*‐value
Sex (M/F)	79/127	73/50	19/18	0.001
Age	71.43 ± 6.26	71.90 ± 7.60	74.08 ± 8.95	0.109
MMSE	28.99 ± 1.18	28.01 ± 1.70	22.78 ± 2.37	<0.001
CDR‐SB	0.04 ± 0.13	1.42 ± 0.88	4.47 ± 1.42	<0.001
ADAS11	5.32 ± 2.73	8.85 ± 3.87	22.61 ± 7.41	<0.001
ADAS13	8.18 ± 4.15	14.11 ± 6.04	33.80 ± 8.72	<0.001
ADASQ4	2.53 ± 1.77	4.64 ± 2.41	9.14 ± 1.25	<0.001
RAVLT immediate	47.24 ± 10.22	36.65 ± 10.12	22.14 ± 6.33	<0.001
RAVLT learning	6.24 ± 2.35	4.55 ± 2.61	1.62 ± 2.02	<0.001
RAVLT forgetting	3.66 ± 2.83	4.83 ± 2.63	4.51 ± 1.37	<0.001
RAVLT perc forgetting	33.47 ± 27.14	57.08 ± 31.56	94.69 ± 13.04	<0.001
APOE4	0.35 ± 0.53	0.55 ± 0.73	1.14 ± 0.67	<0.001

Abbreviations: ADAS, Alzheimer's Disease Assessment Scale–Cognitive Subscales; ADNI, Alzheimer's Disease Neuroimaging Initiative; APOE4, apolipoprotein E4; CDR‐SB, Clinical Dementia Rating Sum of Boxes; MMSE, Mini‐Mental State Examination; RAVLT, Rey Auditory Verbal Learning Test.

### 3.2 Multimodal fusion reveals complementary connectome information and AD‐related alterations

**FIGURE 2:**
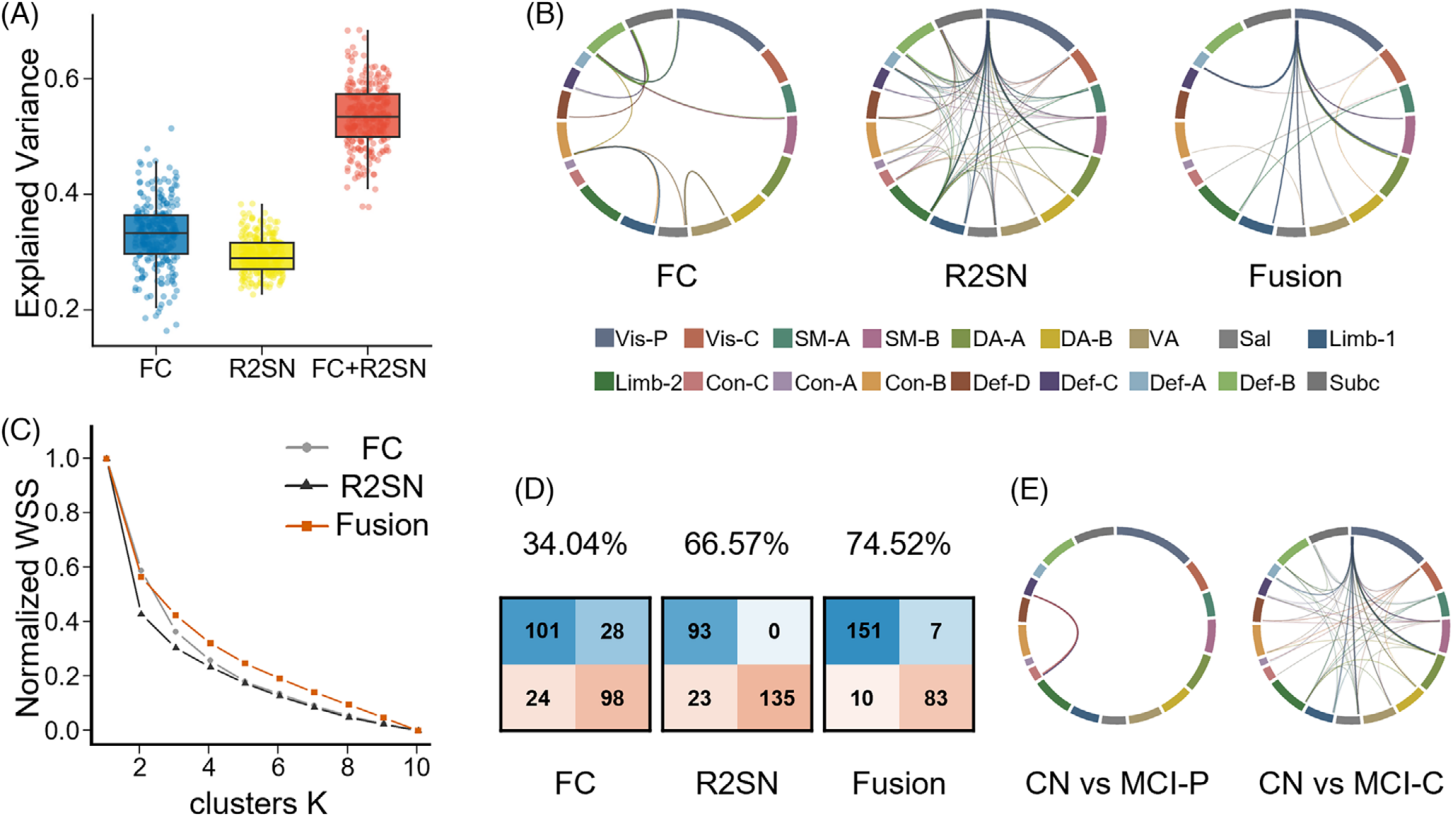
Network integration and clustering results. (A) Adjusted *R* ^2^ values from GAMs evaluating the contributions of structural and functional networks to the fusion network. (B) Chord plots of AD‐related altered connections showing consistency across MCADI and ADNI cohorts. (C) Normalized WSS curves for FC, R2SN, and Fusion networks. (D) ARI Confusion matrices of clustering stability. (E) Distinct topological damage patterns for the two identified subgroups. ADNI, Alzheimer's Disease Neuroimaging Initiative; ARI, adjusted Rand Index; GAMs, generalized additive models; MCADI, Multi‐Center Alzheimer Disease Imaging Consortium; WSS, within‐cluster sum of squares.

Based on the GAM analysis within the CN participants of the MCADI cohort (N = 256), R2SN and FC independently explained 29% ± 3% and 33% ± 6% of the variance in the fused network, respectively. When both modalities were modeled jointly, they accounted for 54% ± 5% of the total variance (Figure 2A). The observation indicates that structural and functional networks convey partially distinct yet complementary topological information to the fused representation. These findings regarding variance decomposition were further replicated and validated in the CN subgroup of the ADNI cohort (Figure S2).

Furthermore, we investigated consistent disease‐related connectome alterations across two cohorts. Only connections showing significant differences in the AD and CN groups in two cohorts were retained, yielding 18 FC connections, 808 R2SN connections, and 68 fused connections (Figure 2B). Cross‐modality overlap analysis revealed no shared altered connections between the unimodal structural and functional networks, indicating largely non‐overlapping disease patterns across modalities. The fused network shared one altered connection with FC and 48 connections with R2SN (accounting for 70.59% of all fused connections) (Figure S3).

### 3.3 Two robust MCI subgroups were identified via the multimodal connectome profiles

To investigate whether the connectome profiles regulated the relationship between pathology burden and clinical expression, we subdivided all MCI groups into two subgroups via the specific connectome profiles. We first evaluated the consistency between clustering solutions. The fusion network demonstrated the highest concordance (74.52%), exceeding structural (66.57%) and functional networks (34.04%) (Figure 2D). Post‐hoc consensus clustering (1000 resampling iterations) further confirmed that these subgroups were robust to data perturbations (Figure S4). To validate the reproducibility of this clustering pattern, we performed external validation in the ADNI cohort. The results demonstrated that the fused network maintained high clustering stability (81.29%), further confirming the cross‐cohort robustness of the multimodal fusion clustering approach (Figure S5).

We further systematically mapped the topological damage patterns of these two clusters. The first subgroup was designated as MCI‐preserved (MCI‐P), whose core feature was limited connection disruption, with an overall network topological structure largely consistent with that of healthy controls. The second subgroup, MCI‐compromised (MCI‐C), exhibited extensive abnormal connectivity changes across multiple brain functional systems, indicating significant impairment of its brain network topological structure (Figure 2E). The topological damage patterns of those two subgroups were consistently validated in the independent ADNI dataset (Figure S5).

### 3.4 MCI subgroups share statistically comparable molecular pathology

**FIGURE 3:**
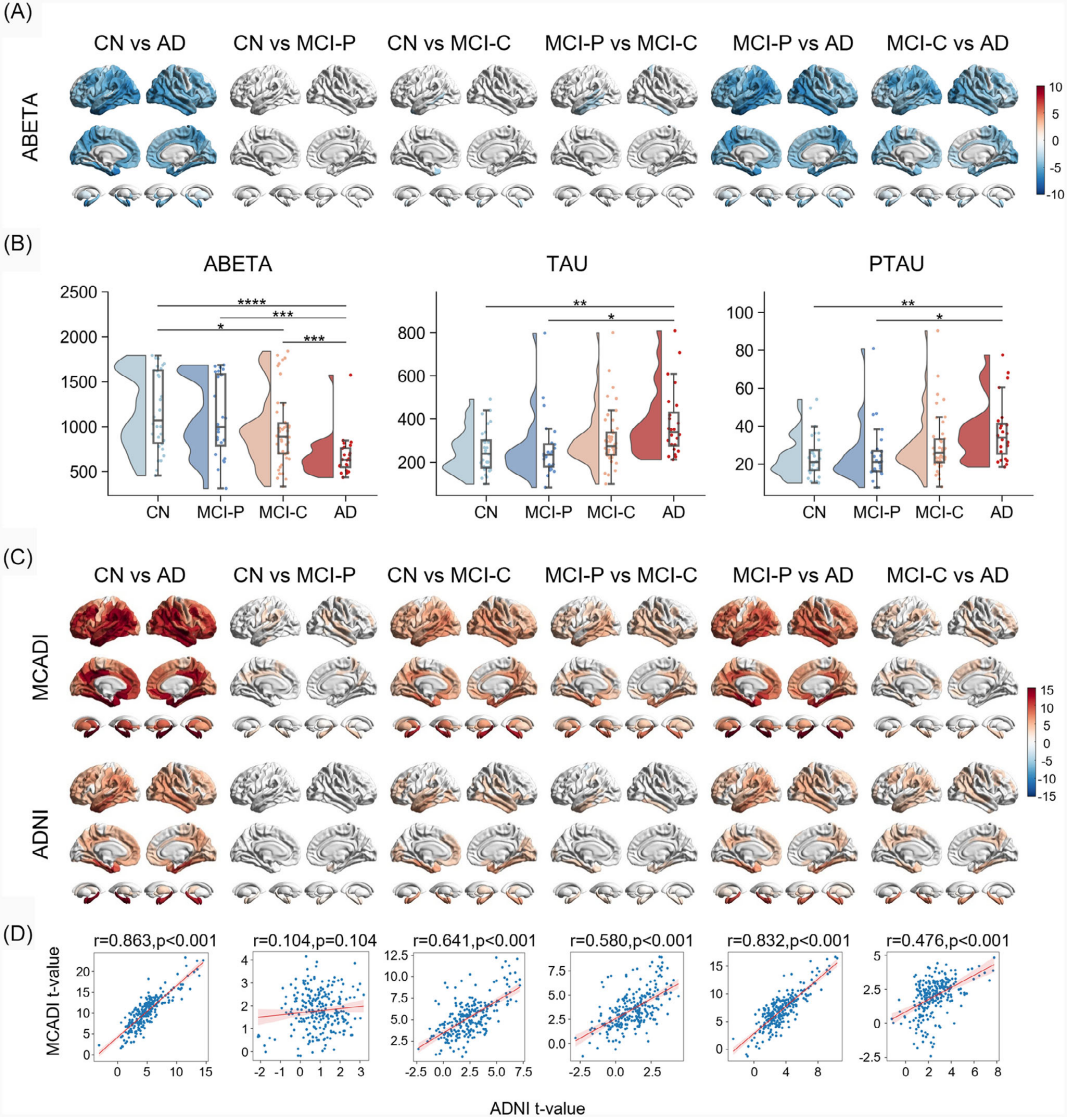
Molecular pathological burden and neurodegenerative alterations across groups. (A) Voxel‐wise T‐maps showing Aβ deposition differences across diagnostic groups. Red and blue indicate positive and negative T‐values, respectively. (B) Comparison of global Aβ, Tau, and p‐Tau levels across groups (**** *p* < 0.0001, *** *p* < 0.001, ** *p* < 0.01, and * *p* < 0.05). (C) Surface‐based cortical maps illustrating regional GMV alterations across group comparisons. The color bar represents the T‐value, where warmer colors indicate regions with significant GMV reductions relative to the baseline or comparison group. (D) Spatial correlation of GMV T‐maps between the discovery (MCADI) and replication (ADNI) cohorts. ADNI, Alzheimer's Disease Neuroimaging Initiative; GMV, gray matter volume; MCADI, Multi‐Center Alzheimer Disease Imaging Consortium.

Surface‐based analyses revealed that the spatial distribution patterns of Aβ deposition were largely overlapping between the MCI‐P and MCI‐C groups, with significant differences confined to only a few brain regions, i.e., the left middle temporal gyrus (Figure 3A). Further group comparisons of global biomarker levels further showed no significant statistical differences in global levels of Aβ, total tau (Tau), or phosphorylated tau (p‐Tau) between the two subgroups (Figure 3B). Stratified subsampling analyses further showed that pathological biomarker differences between MCI‐P and MCI‐C remained largely non‐significant across repeated iterations (Figure S6). These findings suggest that the observed brain network differences occurred against a background of essentially consistent upstream molecular pathology states, ruling out the dominant influence of primary AD pathological differences on network alterations.

### 3.5 MCI subgroups diverge in neurodegeneration and clinical progression

Subsequently, we systematically evaluated the characteristic differences between the two MCI subgroups in terms of downstream brain structure, metabolism, and clinical phenotypes. In the MCADI cohort, regional comparisons of gray matter volume (GMV) revealed more severe anatomical alterations in MCI‐C than MCI‐P (*p* < 0.05, Bonferroni corrected) (Figure 3C). Those spatial distribution patterns were effectively validated in the ADNI cohort (*R* = 0.580, *p* < 0.001), confirming the cross‐cohort robustness of these structural differences (Figure 3D). More importantly, those specific spatial profiles were also found in other neuroimaging biomarkers, that is, cortical thickness (CT) and glucose metabolism (Figure S7).

**FIGURE 4:**
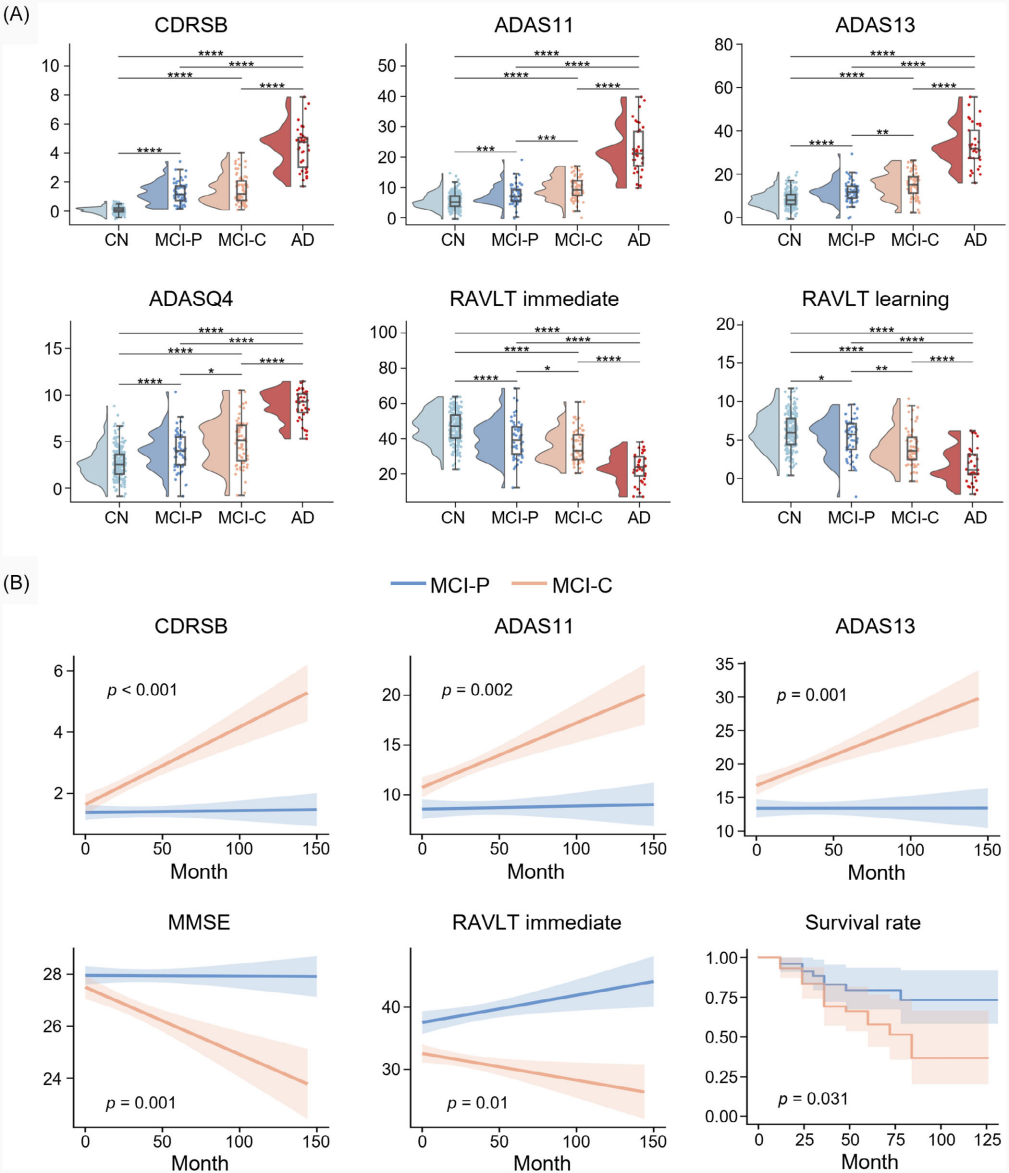
Baseline clinical profiles and longitudinal progression across MCI groups. (A) Baseline cognitive performance across groups, including general cognition (CDR‐SB, ADAS11, ADAS13, ADASQ4) and memory (RAVLT immediate and learning) (**** *p* < 0.0001, *** *p* < 0.001, ** *p* < 0.01, * *p* < 0.05). (B) Predicted progression of cognitive scores over a 150‐month follow‐up based on linear mixed‐effects models. Shaded areas indicate 95% confidence intervals. Kaplan–Meier survival curves showing the probability of remaining dementia‐free. ADAS, Alzheimer's Disease Assessment Scale–Cognitive Subscales; CDR‐SB, Clinical Dementia Rating Sum of Boxes; MCI, mild cognitive impairment; RAVLT, Rey Auditory Verbal Learning Test.

In cognitive ability, the MCI‐C group exhibited significantly worse cognitive performance across multiple domains at baseline, including memory and general cognition (Figure 4A). Permutation tests with 1,000 iterations robustly confirmed the statistical reliability of the intergroup differences in the above cognitive domains except CDR‐SB (Figure S8). In addition, stratified subsampling analyses with 1000 iterations further demonstrated that the intergroup differences in ADAS11, ADAS13, ADASQ4, and RAVLT learning remained reproducible across repeated iterations (Figure S9). Longitudinal mixed‐effects models indicated a faster rate of cognitive decline in MCI‐C relative to MCI‐P. Survival analysis also demonstrated that MCI‐C participants had a dramatically higher probability of progressing to AD compared to MCI‐P (Figure 4B). The intergroup difference in this progression risk was further verified by nonparametric permutation tests and stratified subsampling analyses, further supporting the stability of the observed results (Figures S10–S12).

### 3.6 Genetic analyses reveal the molecular basis of MCI network heterogeneity

**FIGURE 5:**
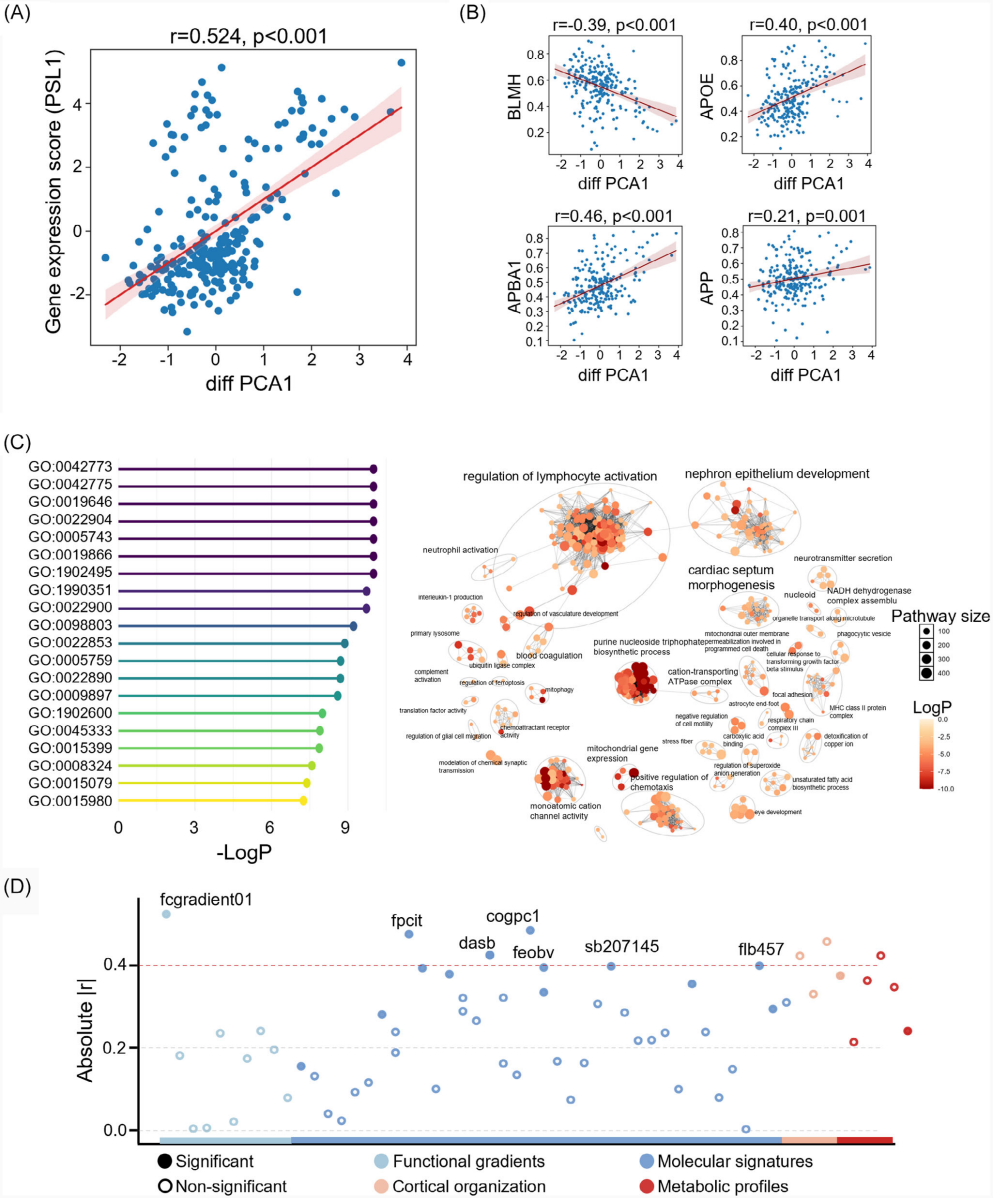
Genetic underpinnings of MCI network heterogeneity. (A) Correlation between the PC1 of differential connections and regional gene expression derived from PLSR regression. (B) Significant correlations between PC1 loadings and regional expression of key AD risk genes. (C) Top 20 significant GO terms and a pathway network of all FDR‐significant terms, summarizing the functional roles of genes ranked by PC1‐associated PLSR weights. (D) Spatial correlations between the PC1 genetic profile and multimodal brain maps. AD, Alzheimer's disease; FDR, false discovery rate; GO, gene ontology; PC1, first principal component; PLSR, partial least squares regression.

To explore the mechanisms by which brain network patterns govern the mapping from pathological burden to cognitive phenotype, we explored the relationship between the connectome profiles and gene expression. The PLSR analysis demonstrated a significant correlation between the PC1 and regional gene expression profiles (*R* = 0.524, *p* < 0.001) (Figure 5A). BrainSMASH‐based spatial autocorrelation‐preserving permutation analyses further confirmed that this association remained significant in both the MCADI and ADNI datasets (Figure S13). Additionally, PC1 exhibited high loadings on several genes previously implicated in AD pathogenesis, including apolipoprotein E (APOE), bleomycin hydrolase (BLMH), amyloid beta precursor protein binding family A member 1 (APBA1), amyloid beta precursor protein binding family A member 2 (APBA2), and amyloid precursor protein (APP) (Figure 5B). Crucially, functional enrichment analysis decoded a coherent biological hierarchy underlying the observed network alterations (Figure 5C). Associated genes were strongly enriched in pathways related to mitochondrial bioenergetics (e.g., membrane permeability and purine nucleoside triphosphate biosynthesis) and synaptic homeostasis (e.g., voltage‐gated cation channel activity). Network clustering further demonstrated a distinct neuroimmune component, involving lymphocyte activation, macrophage migration, and interleukin‑1 production. In addition, BrainSMASH‐based spatial autocorrelation‐preserving permutation analyses revealed significant spatial associations between the PC1 map and several neuromaps‐derived annotations, particularly the principal functional gradient and neurotransmitter‐related molecular signatures, including fpcit, dasb, and cogpc1 (Figure 5D). These findings were robustly validated in the independent ADNI cohort (Figures S14–S15).

## 4 DISCUSSION

**FIGURE 6:**
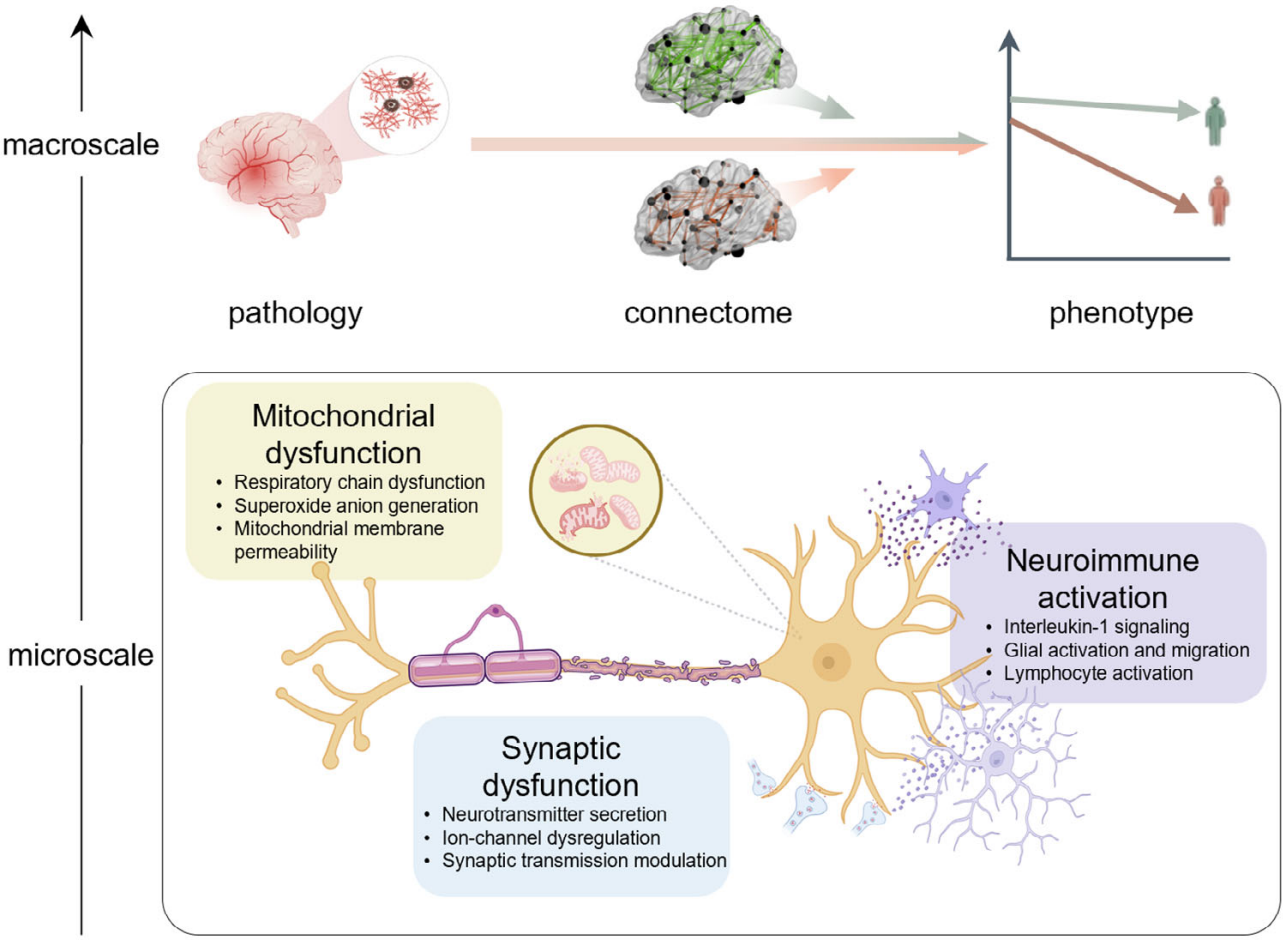
The schematic diagram of connectome‐mediated modulation of the relationship between pathological burden and clinical phenotypes, ranging from the microscale to the macroscale. Created with BioRender.com.

In this study, we demonstrate that the brain connectome acts as a critical active mediator, rather than a mere passive endophenotype, shaping the clinical trajectories of individuals with MCI. Specifically, we identified two biologically distinct subgroups, MCI‐P and MCI‐C, which exhibit markedly divergent downstream clinical outcomes despite sharing comparable upstream molecular pathology. This striking dissociation supports a systems‐level model in which brain networks define a resilience landscape, governing how molecular pathology translates into clinical impairment (Figure 6).

The robust identification of these connectome‐mediated subgroups was made possible by the SNF approach, which successfully synthesized R2SN and FC networks. Crucially, our analysis demonstrated that this fused network is not a mere linear superposition of its constituent modalities; rather, it represents a high‐dimensional synthesis that captures complementary signals across modalities. Furthermore, the fused architecture highly aligned with the principal cortical gradient, particularly the sensorimotor‐association axis, which suggests that R2SN provides a stable anatomical scaffold that fundamentally constrains functional dynamics.^33^, ^34^, ^35^, ^36^, ^37^ By synergizing this structural backbone with dynamic functional information, our multimodal framework yielded markedly larger effect sizes in differentiating neurodegenerative markers, effectively mitigating multi‐center noise and achieving superior clustering stability.

Building upon this robust multimodal framework, data‐driven clustering successfully stratified the cohort into two distinct subgroups: MCI‐P and MCI‐C. These groups exhibited starkly contrasting connectome profiles. While the MCI‐P subgroup maintained preserved multimodal network integrity, the MCI‐C subgroup demonstrated extensive connectome breakdown. Given that these two groups share a comparable burden of AD‐related molecular pathology yet experience highly divergent clinical trajectories, this macroscopic divergence in network topology reveals that the multimodal connectome exerts an active modulatory role — rather than merely reflecting a passive endophenotype — shaping how upstream molecular pathology translates into downstream clinical phenotypes.^38^, ^39^, ^40^ Specifically, the connectome abnormalities observed in MCI‐C were predominantly concentrated within large‐scale visual, somatomotor, limbic, and default mode systems. These systems are broadly involved in sensory integration, motor coordination, memory‐related processing, and internally directed cognition.^41^ In particular, limbic and default mode systems are known to be highly vulnerable to AD‐related neurodegenerative and pathological processes.^12^ Therefore, the preferential disruption of these networks in MCI‐C may provide a systems‐level substrate linking comparable molecular pathology to more severe clinical manifestations. Relatively preserved network organization in MCI‐P may support more efficient large‐scale communication and compensatory processing across distributed systems, thereby buffering the clinical impact of pathological accumulation. Conversely, widespread disruption of multimodal connectome architecture in MCI‐C may impair global communication efficiency and reduce compensatory flexibility, contributing to accelerated neurodegeneration and cognitive decline. Together, these findings support a connectome‐mediated framework of resilience and vulnerability, in which network organization partially modulates the relationship between molecular pathology and clinical expression.

Next, we sought to uncover the microscale biological substrates driving these topological differences. Through imaging‐transcriptomic analyses, we linked the observed fusion network divergence between MCI‐P and MCI‐C to specific regional gene expression profiles. This network disintegration was predominantly associated with transcriptional signatures enriched in three interconnected domains: mitochondrial bioenergetics, synaptic ion transport, and immune‐related processes. These findings suggest a close coupling between molecular vulnerability and large‐scale connectome organization. Efficient large‐scale network communication is highly dependent on metabolic support, synaptic signaling fidelity, and immune homeostasis.^42^, ^43^, ^44^, ^45^ Therefore, disturbances in these biological systems may preferentially compromise highly integrated brain networks and increase vulnerability to large‐scale connectome disruption. Compared with the MCI‐P subgroup, the widespread connectome abnormalities observed in MCI‐C may reflect a brain state characterized by impaired metabolic homeostasis, disrupted synaptic regulation, and increased susceptibility to immune‐related dysfunction, collectively contributing to reduced network stability and resilience. However, the precise causal direction of these associations remains unresolved. It is unclear whether pre‐existing metabolic, synaptic, and immune vulnerabilities contribute to large‐scale connectome disruption, or whether progressive network disintegration itself further exacerbates biological dysfunction during disease progression. Nevertheless, this multiscale convergence provides a biological framework linking microscale molecular vulnerability to macroscale network disruption and heterogeneous clinical trajectories.

This heterogeneity may partially explain the modest and variable treatment effects observed in recent anti‐amyloid trials: the inadvertent inclusion of MCI‐C individuals who have already crossed a threshold of irreversible connectome breakdown — alongside MCI‐P individuals who may remain stable regardless of intervention — could dilute therapeutic signals.^46^ By employing our multimodal profiling as a stratification biomarker, clinicians can shift from reactive observation to proactive, subgroup‐specific intervention. For MCI‐P individuals, strategies could focus on bolstering their existing systems‐level resilience through lifestyle or pharmacological neuroprotection.^47^ For the MCI‐C subgroup, the identification of a compromised network suggests that aggressive multi‐target interventions — addressing not just amyloid, but also the metabolic and immune dysfunctions we identified — may be required before structural integrity reaches a point of no return. Broadly, our framework offers a pathway toward personalized prognosis, enabling the delivery of the right intervention to the right patient at the most effective stage of the disease.

## 5 LIMITATIONS

Several limitations warrant consideration. Our imaging‐genetics analyses relied on the Allen Human Brain Atlas, which is derived from neurotypical adult brains. While this provides a foundational map of spatial gene expression, it does not capture disease‐specific transcriptomic alterations occurring along the AD continuum. In addition, the GSEA findings were based on group‐level spatial transcriptomic associations and should therefore be interpreted as population‐level biological patterns rather than direct individual‐level molecular evidence. Furthermore, pathological biomarkers were only available in a subset of participants, resulting in incomplete molecular characterization and potentially reduced statistical power. Moreover, while our study focused on structural and functional integration, the lack of additional neurobiological modalities may limit a more comprehensive characterization of MCI heterogeneity. Future multimodal studies are needed to better capture these multi‐layered pathological processes.

## AUTHOR CONTRIBUTIONS

Yating Li analyzed and performed the measurements; Shaozhen Yan, Dawei Wang, Hongxiang Yao, Bo Zhou, Jie Lu, Pan Wang, Zhengluan Liao, Ying Han and Xi Zhang collected the data; Yating Li and Kun Zhao were principally responsible for preparing the manuscript; Kun Zhao, YiheZhang, Yongbin Wei, Dong Wang, Rongshen Zhou and Yong Liu revised the manuscript; Kun Zhao, Yihe Zhang and Yong Liu supervised the project.

## CONFLICT OF INTEREST STATEMENT

The authors report no conflicts of interest. Author disclosures are available in the Supporting Information.

## CONSENT STATEMENT

This study was approved by the institutional review board, and all participants provided written informed consent prior to enrollment.

## Supporting information



Supporting Information

## References

[1] Frisoni GB , Aho E , Brayne C , et al. Alzheimer's disease outlook: controversies and future directions. Lancet. 2025;406(10510):1424‐1442. doi:10.1016/S0140-6736(25)01389-3 40997840

[2] 2024 Alzheimer's disease facts and figures. Alzheimers Dement. 2024;20(5):3708‐3821. doi:10.1002/alz.13809 38689398 PMC11095490

[3] Frisoni GB , Hansson O , Nichols E , et al. New landscape of the diagnosis of Alzheimer's disease. Lancet. 2025;406(10510):1389‐1407. doi:10.1016/S0140-6736(25)01294-2 40997838

[4] Jack CR , Bennett DA , Blennow K , et al. NIA‐AA research framework: toward a biological definition of Alzheimer's disease. Alzheimers Dement. 2018;14(4):535‐562. doi:10.1016/j.jalz.2018.02.018 29653606 PMC5958625

[5] Busche MA , Hyman BT . Synergy between amyloid‐beta and tau in Alzheimer's disease. Nat Neurosci. 2020;23(10):1183‐1193. doi:10.1038/s41593-020-0687-6 32778792 PMC11831977

[6] Wei X , Zhang T , Xiong R , et al. Mapping Heterogeneous Brain Structural Subtypes in Alzheimer's Disease and Mild Cognitive Impairment Using Normative Models. Translational Psychiatry; 2026. 10.1038/s41398-026-03902-0 PMC13022452 41771832

[7] Scheltens P , De Strooper B , Kivipelto M , et al. Alzheimer's disease. Lancet. 2021;397(10284):1577‐1590. doi:10.1016/S0140-6736(20)32205-4 33667416 PMC8354300

[8] Brown CA , Mundada NS , Cousins KAQ , et al. Evaluation of copathology and clinical trajectories in individuals with tau‐clinical mismatch. JAMA Neurol. 2026;83(2):126‐136. doi:10.1001/jamaneurol.2025.4974 41396614 PMC12706664

[9] Liu Q , Xiong H , Shi W , et al. Energy inefficiency underpinning brain state dysregulation in individuals with major depressive disorder. Nat Ment Health. 2026;4(3):400‐415. doi:10.1038/s44220-025-00583-4

[10] Yu M , Sporns O , Saykin AJ . The human connectome in Alzheimer disease—relationship to biomarkers and genetics. Nature Reviews Neurology. 2021;17(9):545‐563. doi:10.1038/s41582-021-00529-1 34285392 PMC8403643

[11] Liu W , Zuo C , Chen Li , et al. The whole‐brain structural and functional connectome in Alzheimer's disease spectrum: a multimodal Bayesian meta‐analysis of graph theoretical characteristics. Neuroscience & Biobehavioral Reviews. 2025;174:106174. doi:10.1016/j.neubiorev.2025.106174 40280288

[12] Lee WJ , Brown JA , Kim HR , et al. Regional Aβ‐tau interactions promote onset and acceleration of Alzheimer's disease tau spreading. Neuron. 2022;110(12):1932‐1943 e5. doi:10.1016/j.neuron.2022.03.034 PMC9233123 35443153

[13] Vogel JW , Young AL , Oxtoby NP , et al. Four distinct trajectories of tau deposition identified in Alzheimer's disease. Nat Med. 2021;27(5):871‐881. doi:10.1038/s41591-021-01309-6 33927414 PMC8686688

[14] Stern Y , Arenaza‐Urquijo EM , Bartrés‐Faz D , et al. Whitepaper: defining and investigating cognitive reserve, brain reserve, and brain maintenance. Alzheimer's & Dementia. 2020;16(9):1305‐1311. doi:10.1016/j.jalz.2018.07.219 PMC6417987 30222945

[15] Zhao K , Zheng Q , Che T , et al. Regional radiomics similarity networks (R2SNs) in the human brain: reproducibility, small‐world properties and a biological basis. Netw Neurosci. 2021;5(3):783‐797. doi:10.1162/netn_a_00200 34746627 PMC8567836

[16] Tian X , Peng Y , Liu S , et al. Spontaneous brain regional dynamics contribute to generalizable brain‐behaviour associations. Nat Hum Behav. 2026;10(2):384‐402. doi:10.1038/s41562-025-02332-0 41168426

[17] Seidlitz J , Vasa F , Shinn M , et al. Morphometric similarity networks detect microscale cortical organization and predict inter‐individual cognitive variation. Neuron. 2018;97(1):231‐247 e7. doi:10.1016/j.neuron.2017.11.039 PMC5763517 29276055

[18] Sadikov A , Choi HL , Cai LT , Mukherjee P . Estimating brain similarity networks with diffusion MRI. Hum Brain Mapp. 2025;46(11):e70313. doi:10.1002/hbm.70313 40782044 PMC12335008

[19] Jin D , Wang P , Zalesky A , et al. Grab‐AD: generalizability and reproducibility of altered brain activity and diagnostic classification in Alzheimer's disease. Hum Brain Mapp. 2020;41(12):3379‐3391. doi:10.1002/hbm.25023 32364666 PMC7375114

[20] Shi Y , Wang Z , Chen P , et al. Episodic memory‐related imaging features as valuable biomarkers for the diagnosis of Alzheimer's disease: a multicenter study based on machine learning. Biol Psychiatry Cogn Neurosci Neuroimaging. 2023;8(2):171‐180. doi:10.1016/j.bpsc.2020.12.007 33712376

[21] Li J , Jin D , Li A , et al. ASAF: altered spontaneous activity fingerprinting in Alzheimer's disease based on multisite fMRI. Sci Bull. 2019;64(14):998‐1010. doi:10.1016/j.scib.2019.04.034 36659811

[22] Jack CR , Bernstein MA , Fox NC , et al. The Alzheimer's disease neuroimaging initiative (ADNI): mRI methods. J Magn Reson Imaging. 2008;27(4):685‐691. doi:10.1002/jmri.21049 18302232 PMC2544629

[23] Gaser C , Dahnke R , Thompson PM , Kurth F , Luders E . The Alzheimer's disease neuroimaging I. CAT: a computational anatomy toolbox for the analysis of structural MRI data. GigaScience. 2024;13(13):giae049. doi:10.1093/gigascience/giae049 39102518 PMC11299546

[24] Esteban O , Markiewicz CJ , Blair RW , et al. fMRIPrep: a robust preprocessing pipeline for functional MRI. Nat Methods. 2019;16(1):111‐116. doi:10.1038/s41592-018-0235-4 30532080 PMC6319393

[25] Fan L , Li H , Zhuo J , et al. The human brainnetome atlas: a new brain atlas based on connectional architecture. Cereb Cortex. 2016;26(8):3508‐3526. doi:10.1093/cercor/bhw157 27230218 PMC4961028

[26] Wang Bo , Mezlini AM , Demir F , et al. Similarity network fusion for aggregating data types on a genomic scale. Nat Methods. 2014;11(3):333‐337. doi:10.1038/nmeth.2810 24464287

[27] Zhao K , Zheng Q , Dyrba M , et al. Regional radiomics similarity networks reveal distinct subtypes and abnormality patterns in mild cognitive impairment. Adv Sci. 2022;9(12):e2104538. doi:10.1002/advs.202104538 PMC9036024 35098696

[28] Shen EH , Overly CC , Jones AR . The Allen human brain atlas: comprehensive gene expression mapping of the human brain. Trends Neurosci. 2012;35(12):711‐714. doi:10.1016/j.tins.2012.09.005 23041053

[29] Li J , Seidlitz J , Suckling J , et al. Cortical structural differences in major depressive disorder correlate with cell type‐specific transcriptional signatures. Nat Commun. 2021;12(1):1647. doi:10.1038/s41467-021-21943-5 33712584 PMC7955076

[30] Zeng H , Shen EH , Hohmann JG , et al. Large‐scale cellular‐resolution gene profiling in human neocortex reveals species‐specific molecular signatures. Cell. 2012;149(2):483‐496. doi:10.1016/j.cell.2012.02.052 22500809 PMC3328777

[31] Markello RD , Hansen JY , Liu Z‐Qi , et al. neuromaps: structural and functional interpretation of brain maps. Nat Methods. 2022;19(11):1472‐1479. doi:10.1038/s41592-022-01625-w 36203018 PMC9636018

[32] Subramanian A , Tamayo P , Mootha VK , et al. Gene set enrichment analysis: a knowledge‐based approach for interpreting genome‐wide expression profiles. Proc Natl Acad Sci U S A. 2005;102(43):15545‐15550. doi:10.1073/pnas.0506580102 16199517 PMC1239896

[33] Pang JC , Aquino KM , Oldehinkel M , et al. Geometric constraints on human brain function. Nature. 2023;618(7965):566‐574. doi:10.1038/s41586-023-06098-1 37258669 PMC10266981

[34] Sydnor VJ , Larsen B , Seidlitz J , et al. Intrinsic activity development unfolds along a sensorimotor‐association cortical axis in youth. Nat Neurosci. 2023;26(4):638‐649. doi:10.1038/s41593-023-01282-y 36973514 PMC10406167

[35] Fotiadis P , Parkes L , Davis KA , Satterthwaite TD , Shinohara RT , Bassett DS . Structure‐function coupling in macroscale human brain networks. Nat Rev Neurosci. 2024;25(10):688‐704. doi:10.1038/s41583-024-00846-6 39103609

[36] Sun Y , Wang P , Zhao K , et al. Structure‐function coupling reveals the brain hierarchical structure dysfunction in Alzheimer's disease: a multicenter study. Alzheimers Dement. 2024;20(9):6305‐6315. doi:10.1002/alz.14123 39072981 PMC11497717

[37] Valk SL , Xu T , Paquola C , et al. Genetic and phylogenetic uncoupling of structure and function in human transmodal cortex. Nat Commun. 2022;13(1):2341. doi:10.1038/s41467-022-29886-1 35534454 PMC9085871

[38] Negro D , Opazo P . Cognitive resilience in Alzheimer's disease: from large‐scale brain networks to synapses. Brain Commun. 2024;6(1):fcae050. doi:10.1093/braincomms/fcae050 38425748 PMC10903981

[39] Boyle R , Connaughton M , McGlinchey E , et al. Connectome‐based predictive modelling of cognitive reserve using task‐based functional connectivity. Eur J Neurosci. 2023;57(3):490‐510. doi:10.1111/ejn.15896 36512321 PMC10107737

[40] Bocancea DI , van Loenhoud AC , Groot C , Barkhof F , van der Flier WM , Ossenkoppele R . Measuring resilience and resistance in aging and Alzheimer disease using residual methods: a systematic review and meta‐analysis. Neurology. 2021;97(10):474‐488. doi:10.1212/WNL.0000000000012499 34266918 PMC8448552

[41] Smallwood J , Bernhardt BC , Leech R , Bzdok D , Jefferies E , Margulies DS . The default mode network in cognition: a topographical perspective. Nat Rev Neurosci. 2021;22(8):503‐513. doi:10.1038/s41583-021-00474-4 34226715

[42] MacMullen C , Sharma N , Davis RL . Mitochondrial dynamics and bioenergetics in Alzheimer's induced pluripotent stem cell‐derived neurons. Brain. 2025;148(4):1405‐1420. doi:10.1093/brain/awae364 39513728 PMC12168126

[43] Bhoi R , Mitra T , Tejaswi K , Manoj V , Ghatak S . Role of ion channels in Alzheimer's disease pathophysiology. J Membr Biol. 2025;258(3):187‐212. doi:10.1007/s00232-025-00341-8 40310500 PMC12081594

[44] Anand C , Abdelnour F , Sipes B , et al. Selective vulnerability and resilience to Alzheimer's disease tauopathy as a function of genes and the connectome. Brain. 2025;148(10):3679‐3693. doi:10.1093/brain/awaf179 40631882 PMC12493037

[45] Rickenbach C , Mallone A , Häusle L , et al. Altered T‐cell reactivity in the early stages of Alzheimer's disease. Brain. 2025;148(9):3364‐3378. doi:10.1093/brain/awaf167 40320887 PMC12404719

[46] Sims JR , Zimmer JA , Evans CD , et al. Donanemab in early symptomatic Alzheimer disease: the TRAILBLAZER‐ALZ 2 randomized clinical trial. JAMA. 2023;330(6):512‐527. doi:10.1001/jama.2023.13239 37459141 PMC10352931

[47] Livingston G , Huntley J , Liu KY , et al. Dementia prevention, intervention, and care: 2024 report of the Lancet standing commission. Lancet. 2024;404(10452):572‐628. doi:10.1016/S0140-6736(24)01296-0 39096926

