# Supplementary material for: Human brain connectome profiles mediate the relationship between pathology burden and clinical phenotypes in Alzheimer's disease
Source: Alzheimers Dement. 2026 Jun 30;22(7):e71638. doi: 10.1002/alz.71638 (PMC13319412; doi:10.1002/alz.71638)
Supplement: Supplementary file 1 — Supporting Information [file ALZ-22-e71638-s001.pdf]

Supplementary Material

Outline

Supplementary Material 1

Methods S1. Subject Information in MCADI 3

Methods S2. Subject Information in ADNI 9

Supplementary figures 11

Figure S1. Parameter stability analysis of the similarity network fusion (SNF) process. 11

Figure S2. Variance decomposition of the fusion network in the ADNI cohort. 12

Figure S3. Overlap of disease-related connectome alterations. 13

Figure S4. Consensus clustering for stability assessment. 14

Figure S5. Clustering stability and topological patterns in ADNI. 15

Figure S6. Robustness of pathological biomarker comparisons across stratified resampling analyses. 16

Figure S7. Validation of cortical thickness and metabolism. 17

Figure S8. Permutation testing of clinical and cognitive differences. 18

Figure S9. Robustness of cognitive comparisons across stratified resampling analyses. 19

Figure S10. Robustness of longitudinal subgroup differences across stratified resampling analyses. 20

Figure S11. Permutation test for survival analysis. 21

Figure S12. Robustness of progression-risk estimation across stratified resampling analyses. 22

Figure S13. Spatial autocorrelation-preserving validation of imaging-transcriptomic

associations. 23

Figure S14. Genetic underpinnings of MCI network heterogeneity in the ADNI dataset. .. 24

Figure S15. Cross-dataset reproducibility of neuromaps association patterns. 25

References 26

Supplementary methods

Methods S1. Subject Information in MCADI

MCADI datasets were used in our previous studies to evaluate altered spontaneous activity in Alzheimer's disease (AD). To minimize the variation in multicenter data, we included the following quality controls: (1) exclusion of subjects with poor fMRI data quality, subjects without complete demographic information, and subjects without Mini-Mental State Examination (MMSE) scores; (2) removal of subjects with large head motion in any direction corresponding to >3 mm or any rotation >3°; and (3) matching the age and gender of the subjects at each center. Here, with permission, we have rewritten the information to maintain the integrity of the present study.

PL_G and PL_S

This study was approved by the Medical Ethics Committee of PLA General Hospital. Written informed consent was obtained from each enrolled subject or his/her authorized guardian. All of the participants were recruited by an advertisement (http://www.301ad.com.cn, Chinese version). Prior to selection for this study, all of the participants were given free physical, psychological and laboratory examinations. All patients received professional suggestions for further treatment.

All of the subjects were right-handed and underwent a battery of neuropsychological tests, including the Mini-Mental State Examination (MMSE), the auditory verbal learning test (AVLT), Geriatric Depression Scale (GDS) [(1)] , Clinical Dementia Rating (CDR) [9] and Activities of Daily Living (ADL) Scale. In brief, the AVLT consisted of 1 learning trial in which a list of 10 Chinese double-character words was read, and the subject was asked to immediately recall as many items as possible. The trial was repeated twice, and the immediate recall score was the average of 3 accurate recalls. After a

5-minute delay, each subject was asked to recall the words from the initial list (AVLT-delayed recall). The subjects were then told to identify the 10 studied words that were inter-mixed with 10 novel words (AVLT-recognition).

The recruited AD patients fulfilled the following inclusion criteria: (1) diagnosed using the National Institute of Neurological and Communicative Disorders and Stroke and the Alzheimer Disease's and Related Disorders Association criteria for probable AD; (2) CDR = 1 or 2; (3) currently receiving no tropic drugs, such as cholinesterase inhibitors; and (4) able to perform the neuropsychological test and tolerate MR scanning.

The diagnostic criteria for MCI were determined as previously described and included the following: (1) memory complaints lasting at least 6 months; (2) CDR = 0.5; (3) intact functional status and ADL< 26; and (4) lack of dementia. The criteria for NC included the following: (1) normal physical status; (2) CDR = 0; and (3) without memory complaints.

The following exclusion criteria were used in this study: (1) metabolic conditions such as hypothyroidism or vitamin B12/folic acid deficiencies; (2) psychiatric disorders such as schizophrenia or depression; (3) infarction or brain hemorrhaging, as indicated by MR/CT imaging; and (4) Parkinsonian syndrome, epilepsy and other nervous system diseases that can influence cognitive function. In addition, patients with a metallic foreign body, such as a cochlear implant, heart stent or other relevant MR scanning contraindications, were excluded from the study.

Related publications can be found elsewhere [(4-13)].

HH_Z

The dataset followed the same protocol as PL_G and PL_S. This study was approved by the Medical

Ethics Committee of Tianjin Huanhu Hospital. The patients were recruited from the memory clinic of the Neurology Department of Tianjin Huanhu Hospital, Tianjin, China. The control subjects were recruited from the local community using advertisements. Written informed consent was obtained from each enrolled subject or his/her authorized guardian. The participants underwent general physical, psychological and laboratory examinations prior to enrollment in the formal study. The participants didn't undergo the auditory verbal learning test. The participants did not take medications that might have influenced cognition during the scans, and all patients received professional suggestions for further treatment.

QL_W

The dataset followed the same protocol as PL_G and PL_S. This study was approved by the Medical Ethics Committee of Qilu Hospital of Shandong University. The patients were recruited from the memory clinic of the Department of Neurology and Radiology, Qilu Hospital of Shandong University, Ji'nan, China. The control subjects were recruited from the local community using advertisements. Written informed consent was obtained from each enrolled subject or his/her authorized guardian. The participants underwent general physical, psychological and laboratory examinations prior to enrollment in the formal study. The participants did not take medications that might have influenced cognition during the scans, and all patients received professional suggestions for further treatment.

XW_H

The study was approved by the Medical Research Ethics Committee and Institutional Review Board of Xuanwu Hospital (ClinicalTrials.gov identifiers: NCT02353884 and NCT02225964). Part of the

data have been used in several previous studies, and detail information can be found elsewhere [14,15].

All subjects underwent a series of standardized clinical evaluations, including a medical history interview, neurologic examination, and a battery of neuropsychological tests. The neuropsychological tests included the Chinese version of the MMSE, the Beijing version of MoCA [16], the CDR [2], the AVLT [17], an ADL assessment, the Hachinski Ischemic Scale, the Hamilton Depression Rating Scale (HAMD) [18], and The Center for Epidemiologic Studies Depression Scale [19]. Confirmation of diagnosis for all subjects was made by the consensus of at least two experienced neurologists in the Neurology Department of Xuanwu Hospital. The diagnoses were based on the available data from the neuropsychological assessment evaluation, a battery of general neurological examinations, and subject symptoms as well as functional capacity reports.

The inclusion criteria for aMCI diagnosis included the following [20]: (a) memory complaints, confirmed by an informant; (b) objectively impaired memory confirmed by neuropsychological tests; (c) a definite history of cognitive decline; (d) not meeting the criteria for dementia according to the Diagnostic and Statistical Manual of Mental Disorders, Fourth Edition, Revised (DSM-IV-R); and (e) a CDR score of 0.5.

AD subjects were diagnosed according to the National Institute of Aging-Alzheimer's Association (NIA-AA) criteria for clinically probable AD [21,22]: (a) meeting the criteria for dementia; (b) insidious and gradual onset (not sudden) over more than 6 months; (c) definite history of declining cognition; (d) initial and most prominent cognitive deficits evident in amnestic or non-amnestic performance; and (e) hippocampal atrophy confirmed by structural MRI.

XW_Z

The NC patients were required to meet the following research criteria: (a) no memory concerns; (b) MMSE and MoCA scores within the normal range (adjusted for age, sex, and education); and (c) a CDR score of 0.

The exclusion criteria applied to all subjects included the following: (a) vascular cognitive impairment (Hachinski Ischemic Scale score > 4 points); (b) severe depression (HAMD score > 24 points or The Center for Epidemiological Studies Depression Scale score > 21 points); (c) other central nervous system diseases that could cause cognitive decline (e.g., epilepsy, brain tumors, Parkinson’s disease, or encephalitis); (d) systemic diseases that could cause cognitive impairments (e.g., anthracemia, syphilis, thyroid dysfunctions, severe anemia, or HIV); (e) a history of psychosis or congenital mental growth retardation; (f) severe hypopsia or dysacusis; (g) cognitive decline caused by traumatic brain injury; (h) severe end-stage disease or severe diseases in acute stages; (i) a history of stroke; or (j) unable to complete neuropsychological tests or with a contraindication for MRI.

All the participants were recruited by advertisement and supported throughout the testing procedures in a specialist neuropsychological research facility at Xuanwu Hospital, Beijing, China. Patients and informants (usually a family member) were interviewed clinically by a senior psychiatrist (X. Zhang). Written consent forms were obtained from all subjects or their legal guardians (usually a family member). The study was approved by the Ethics Committee of Xuanwu Hospital. AD subjects were diagnosed using standard operationalized criteria (DSM-IVR [American Psychiatric Association, 1994] and NINCDS-ADRDA [21]]).

The inclusion criteria for AD diagnosis included the following: severity of dementia was assessed

using the Clinical Dementia Rating (CDR) scale [28]. Patients with a diagnosis of AD and CDR score of 1 were classified as mild AD; patients with a CDR score of 2 or 3 were diagnosed as severe AD.

Mild cognitive impairment (MCI) was diagnosed according to standard criteria [3,23,24], which included subjective memory loss with objective evidence of memory impairment in the context of normal or near-normal performance on other domains of cognitive functioning; minimal impairment of activities of daily living; and a CDR score of 0.5. Normal volunteers have a CDR score of 0.

All participants satisfied the following inclusion criteria: (1) no history of an affective disorder within one month prior to assessment; (2) normal vision and audition; (3) able to cooperate with cognitive testing; (4) aged between 50 and 90 years; (5) no clinical history of stroke or other severe cerebrovascular disease; and (6) no more than one lacunar infarction, without patchy or diffuse leukoaraiosis, on neuroradiological assessment of conventional MR images.

The exclusion criteria included the following: (1) severe general medical disorders of cardiovascular, endocrine, renal or hepatic systems; neurological disorders associated with potential cognitive dysfunction, including local brain lesions, traumatic brain injury with loss of consciousness or confusion, and dementia associated with neurosyphilis, Parkinsonism or Lewy body disease; psychiatric disorders including depression, alcohol or drug abuse; (2) concomitant use of psychotropic medication in a large quantity; and (3) insufficient cognitive capacity to understand and cooperate with study procedures.

All patients underwent a complete physical and neurological examination, an extensive battery of neuropsychological assessments, and standard laboratory tests. Healthy volunteers underwent a brief clinical interview and MMSE to confirm that they satisfied the exclusion criteria for cognitive

deficits, psychoactive drug use, and clinical disorders.

Methods S2. Subject Information in ADNI

The Alzheimer's Disease Neuroimaging Initiative (ADNI)(http://adni.loni.usc.edu), launched in 2003 as a public-private partnership and is a longitudinal natural history study whose primary purpose is to inform the design of therapeutic trials in Alzheimer's disease (AD).

Briefly, the NC patients were required to meet the following inclusion criteria: 1) No memory complaints; 2) Normal memory documented by scoring below education-adjusted cutoffs on the Logical Memory II subscale (Delayed Paragraph Recall, Paragraph A only) from the Wechsler Memory Scale-Revised; 3) MMSE score between 24 and 30 inclusive (Exceptions may be made for participants with less than 8 years of education at the discretion of the Project Director); 4) Clinical Dementia Rating = 0. Memory Box score must be at least 0. Inclusion of MCI in ADNI was mainly included: 1) Participant must express a subjective memory concern; 2) Abnormal memory function documented by scoring below education-adjusted cutoffs on the Logical Memory II subscale (Delayed Paragraph Recall, Paragraph A only) from the Wechsler Memory Scale-Revised; 3) MMSE score between 24 and 30 inclusive (Exceptions may be made for participants with less than 8 years of education at the discretion of the Project Director); 4) Clinical Dementia Rating = 0.5. Memory Box score must be at least 0.5; 5) General cognition and functional performance sufficiently preserved such that a diagnosis of Alzheimer's disease cannot be made by the site physician at the time of the Screening Visit. Inclusion of AD in ADNI was mainly included: 1) Participant must express a subjective memory concern; 2) Abnormal memory function documented by scoring below education-adjusted cutoffs on the Logical Memory II subscale (Delayed Paragraph Recall, Paragraph

A only) from the Wechsler Memory Scale-Revised; 3) MMSE score between 20 and 24 inclusive (Exceptions for scores of 24 and 25 may be made for participants with less than 8 years of education at the discretion of the Project Director); 4) Clinical Dementia Rating = 0.5 or 1.0; 5) NINCDS/ADRDA criteria for probable AD.

The exclusion criteria in ADNI mainly included: 1) Any significant neurologic disease other than Alzheimer's disease; 2) Screening/Baseline MRI brain scan with evidence of infection, infarction, or other focal lesions or multiple lacunes or lacunes in a critical memory structure; 3) Subjects that have any contraindications for MRI studies, including the presence of cardiac pacemakers, or metal fragments or foreign objects in the eyes, skin or body; 4) Major depression, bipolar disorder as described in DSM-IV within the past 1 year. Psychotic features, agitation, or behavioral problems within the last 3 months that could lead to difficulty complying with the protocol. The completed inclusion and exclusion criteria can be found in the Clinical Protocols in ADNI (adni.loni.usc.edu/methods/documents/).

Both structural MRI and resting-state fMRI images were acquired on 3.0-Tesla scanners. Resting-state functional images were obtained by using echo-planar imaging (EPI) sequence. This detailed information can be found in MRI Protocols in ADNI (adni.loni.usc.edu/methods/documents/) elsewhere in our previous studies [(25-31)] .

Supplementary figures

Figure S1. Parameter stability analysis of the similarity network fusion (SNF) process.

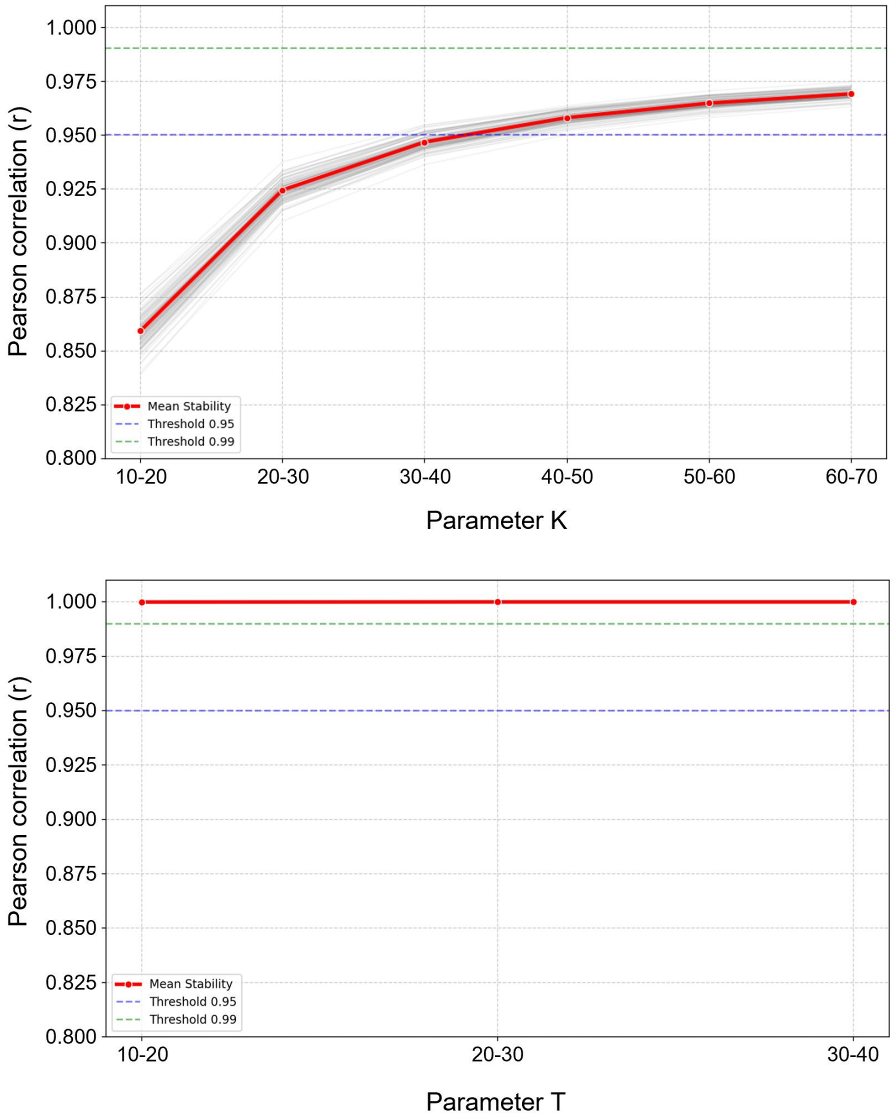

Figure S1. Parameter stability analysis of the similarity network fusion (SNF) process. Line graphs illustrating the step-wise consistency (Pearson correlation, r) of the fusion network evaluated across varying parameter transitions. The red solid line represents the mean stability, while the light gray lines indicate individual variations. The blue and green dashed lines represent the reference stability thresholds of 0.95 and 0.99, respectively.

Figure S2. Variance decomposition of the fusion network in the ADNI cohort.

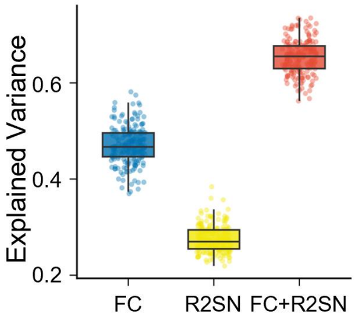

Figure S2. Variance decomposition of the fusion network in the ADNI cohort. Boxplots showing the proportion of variance in the fusion network explained independently and jointly by FC and R2SN within the CN subgroup.

Figure S3. Overlap of disease-related connectome alterations.

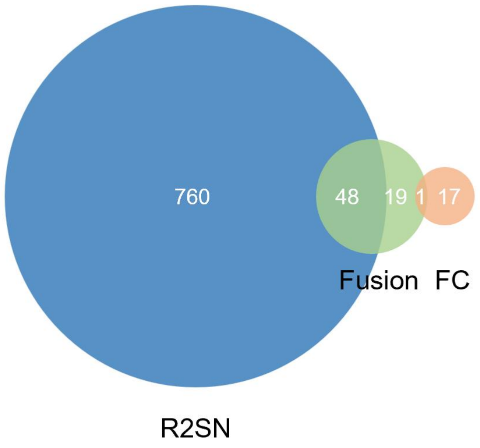

Figure S3. Overlap of disease-related connectome alterations. Venn diagram showing shared and unique altered connections among R2SN, FC, and fusion networks.

Figure S4. Consensus clustering for stability assessment.

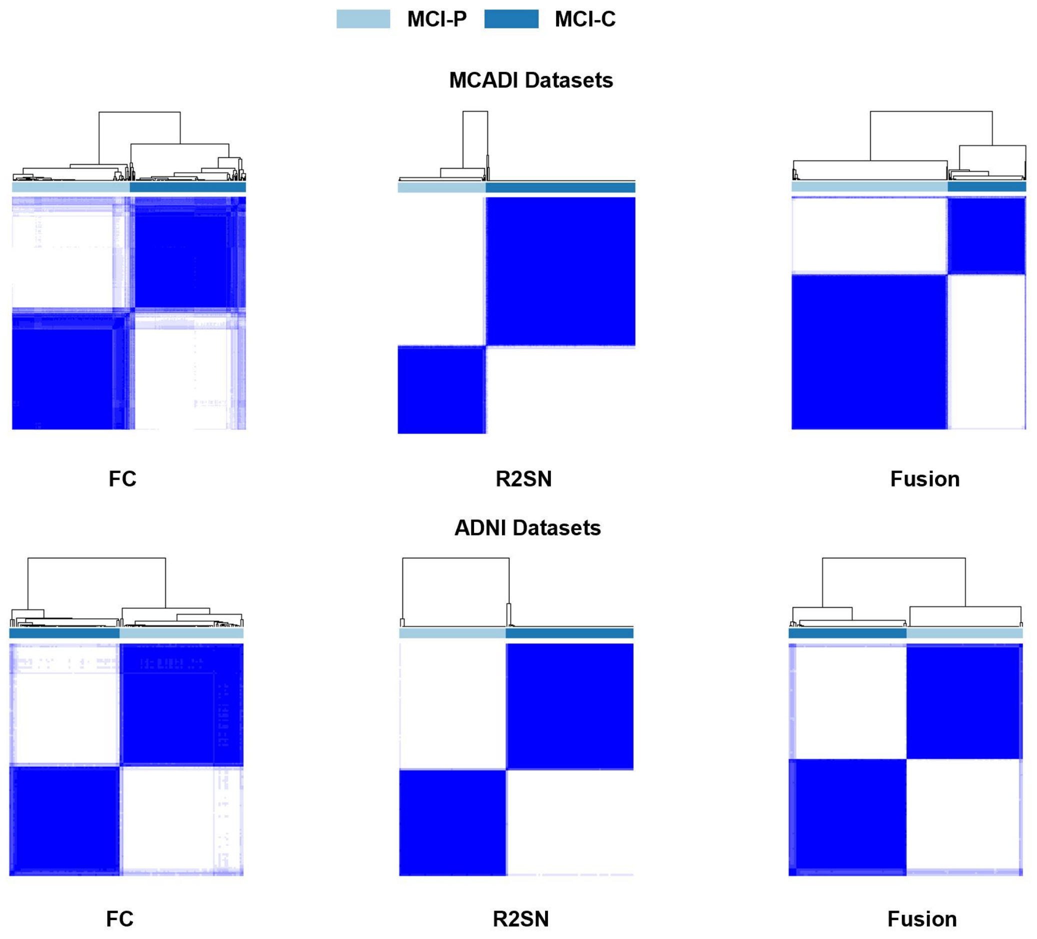

Figure S4. Consensus clustering for stability assessment. Consensus matrices for ADNI and MCADI datasets across FC, R2SN, and fusion networks (K=2, 1,000 iterations). Deep blue and white indicate high and zero co-occurrence frequency, respectively.

Figure S5. Clustering stability and topological patterns in ADNI.

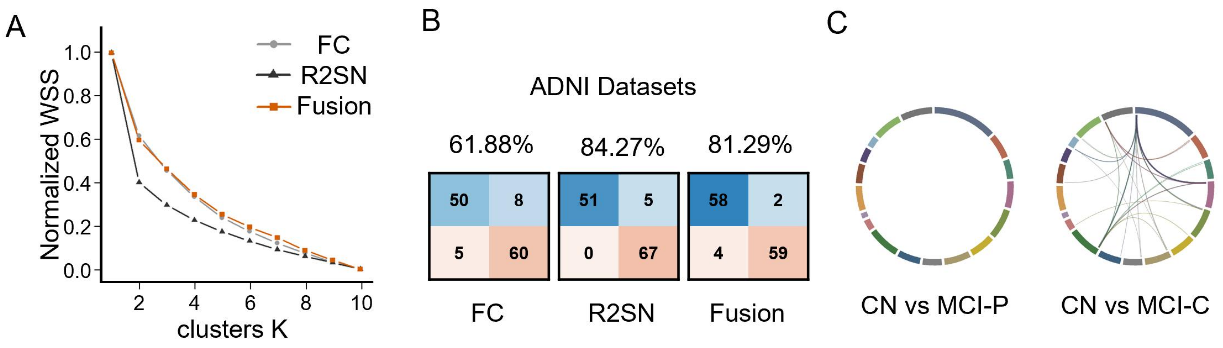

Figure S5. Clustering stability and topological patterns in ADNI. A) Normalized WSS across cluster numbers for FC, R2SN, and fusion networks. B) Adjusted Rand Index (ARI) Confusion matrices of clustering stability. C) Distinct topological damage patterns for the two identified subgroups.

Figure S6. Robustness of pathological biomarker comparisons across stratified resampling analyses.

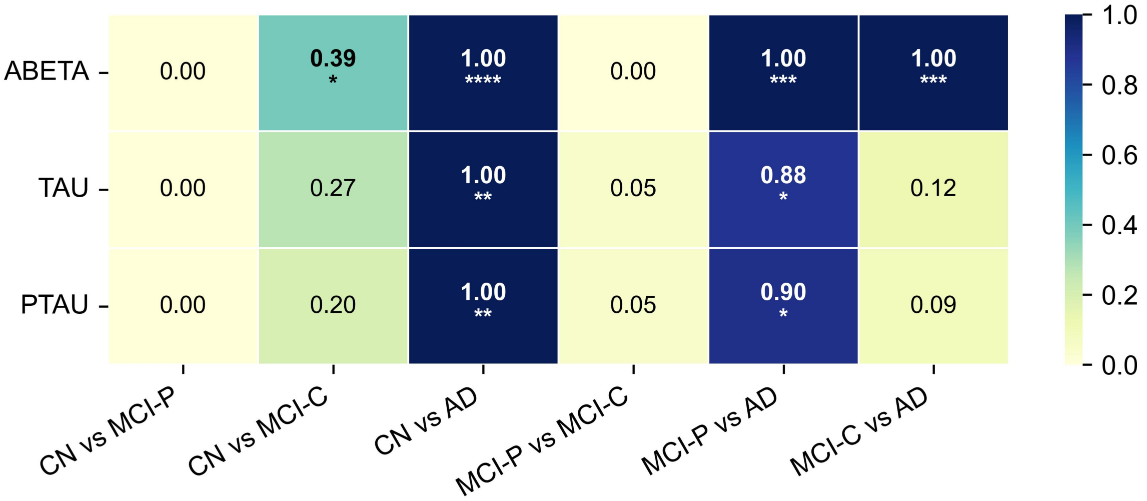

Figure S6. Robustness of pathological biomarker comparisons across stratified resampling analyses. Heatmaps show the reproducibility rate of significant group differences across 1,000 stratified subsampling iterations for pathological biomarkers.

Figure S7. Validation of cortical thickness and metabolism.

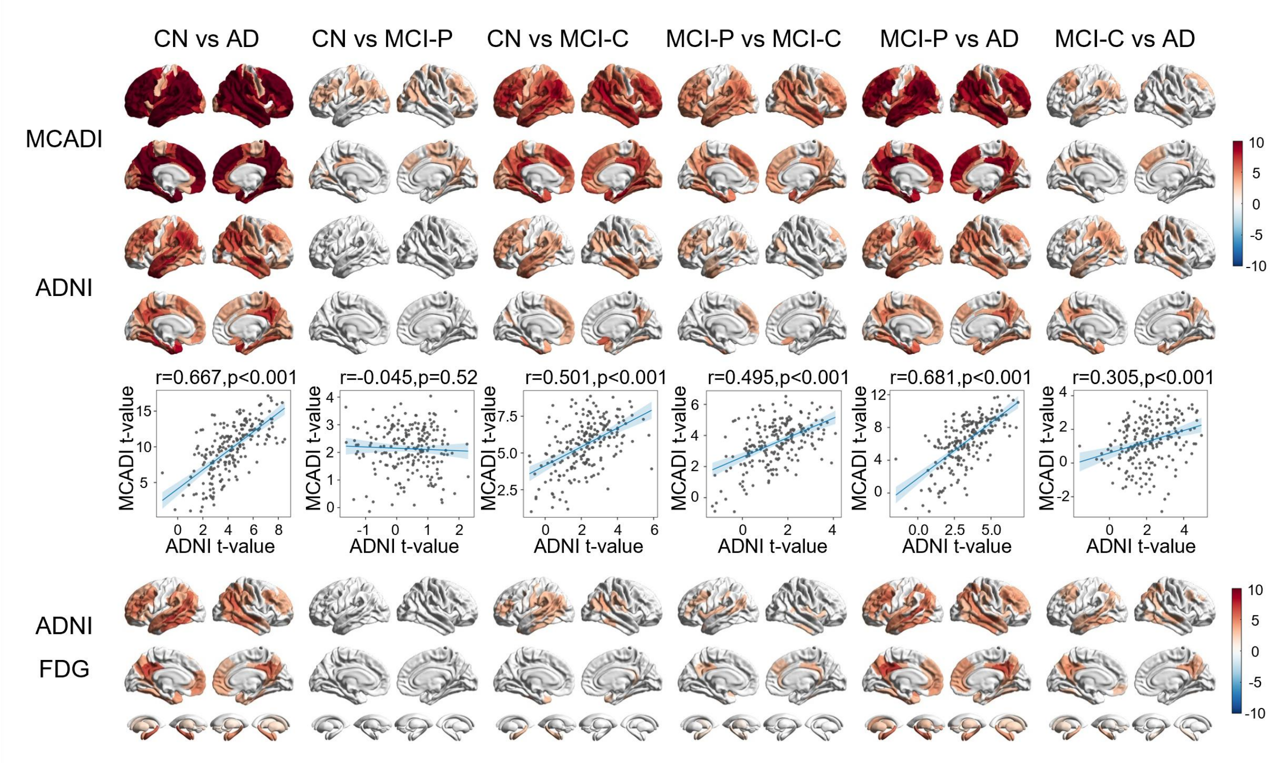

Figure S7. Validation of cortical thickness and metabolism. Surface-based brain maps illustrating differences in cortical thickness (CT) and fluorodeoxyglucose (FDG) metabolism among CN, AD, MCI-P, and MCI-C groups in the MCADI and ADNI cohorts. The color bar indicates the T-value. Scatter plots demonstrate the spatial correlation of T-values between the MCADI and ADNI cohorts for each corresponding pairwise comparison.

Figure S8. Permutation testing of clinical and cognitive differences.

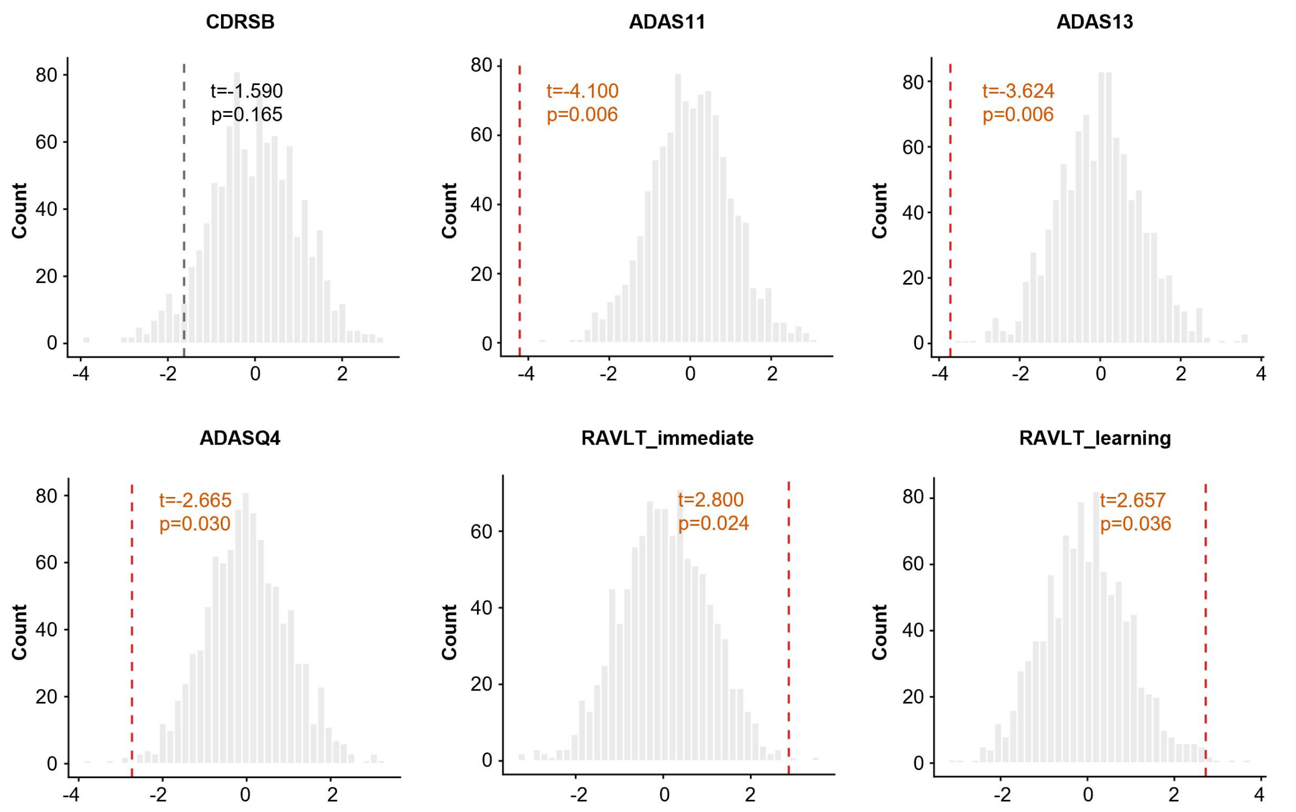

Figure S8. Permutation testing of clinical and cognitive differences. Null distributions (1,000 iterations) evaluating the statistical reliability of intergroup differences across cognitive domains. Dashed orange lines indicate observed statistics for comparisons among MCI-P and MCI-C.

Figure S9. Robustness of cognitive comparisons across stratified resampling analyses.

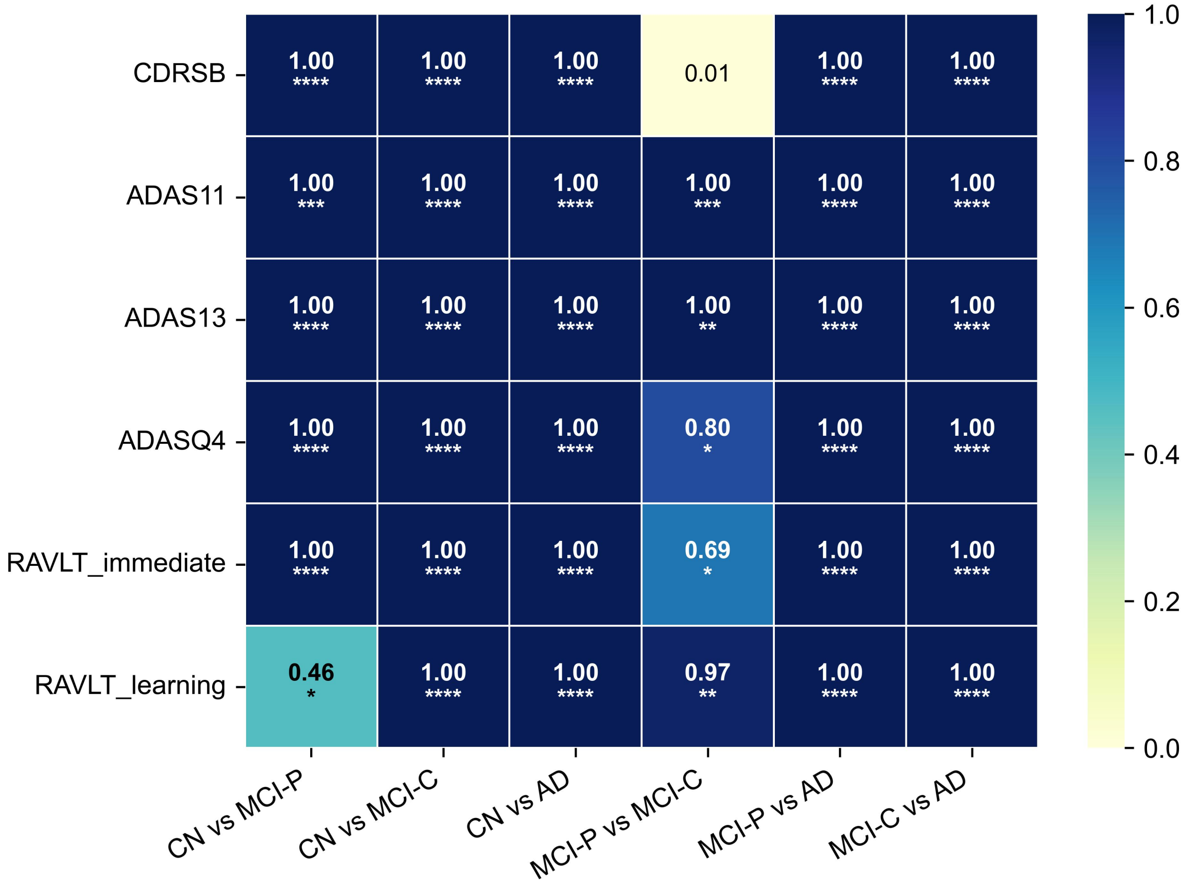

Figure S9. Robustness of cognitive comparisons across stratified resampling analyses. Heatmaps show the reproducibility rate of significant group differences across 1,000 stratified subsampling iterations for cognitive measures.

Figure S10. Robustness of longitudinal subgroup differences across stratified resampling analyses.

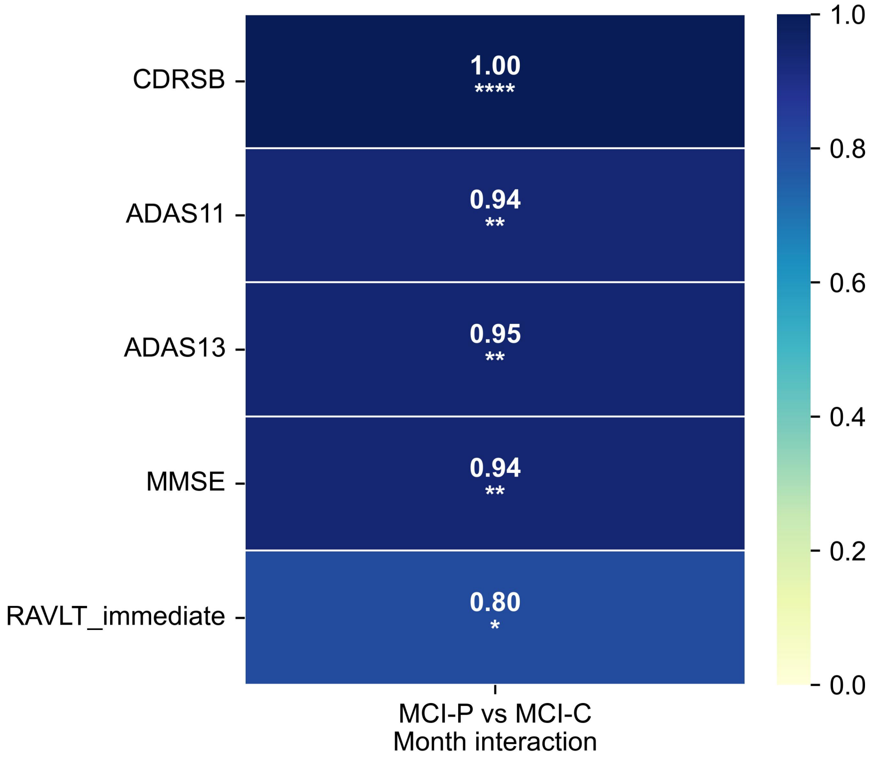

Figure S10. Robustness of longitudinal subgroup differences across stratified resampling analyses. Heatmap shows the reproducibility rate of significant subgroup-by-time interaction effects across 1,000 stratified subsampling iterations for longitudinal cognitive trajectories.

Figure S11. Permutation test for survival analysis.

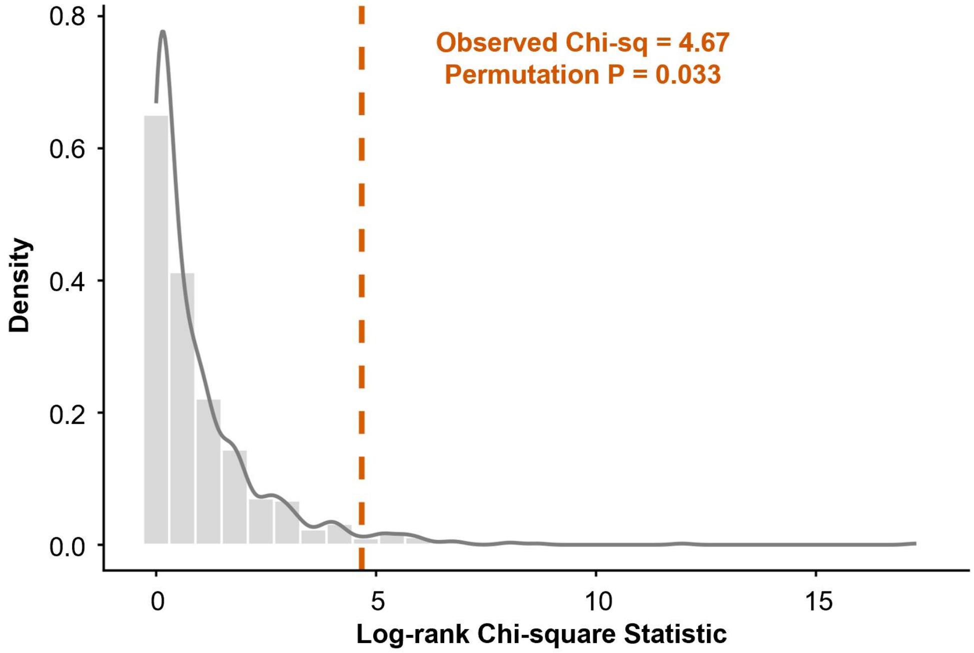

Figure S11. Permutation test for survival analysis. Null distribution (1,000 iterations) with the vertical dashed line marking the observed Log-rank Chi-square statistic (χ^2= 4.67, P = 0.033).

Figure S12. Robustness of progression-risk estimation across stratified resampling analyses.

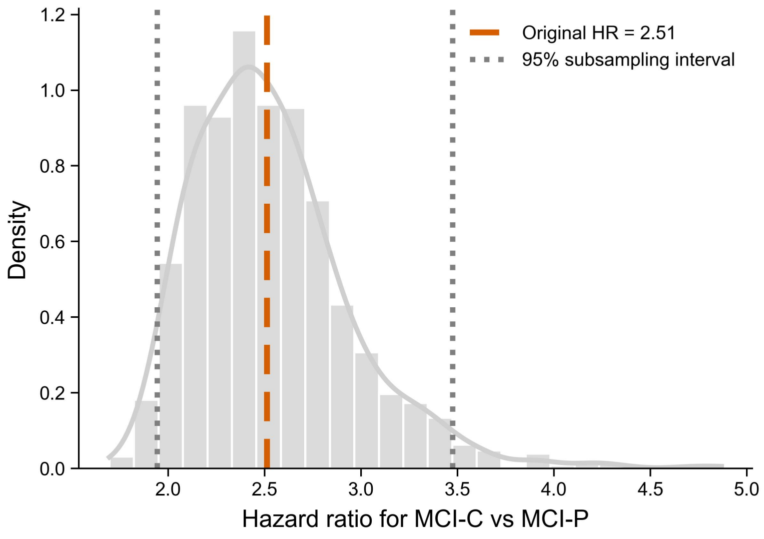

Figure S12. Robustness of progression-risk estimation across stratified resampling analyses. Distribution of hazard ratios obtained from 1,000 stratified subsampling Cox regression analyses. The orange dashed line indicates the original hazard ratio estimated from the full cohort, and the gray dotted lines indicate the 95% resampling interval.

Figure S13. Spatial autocorrelation-preserving validation of imaging-transcriptomic associations.

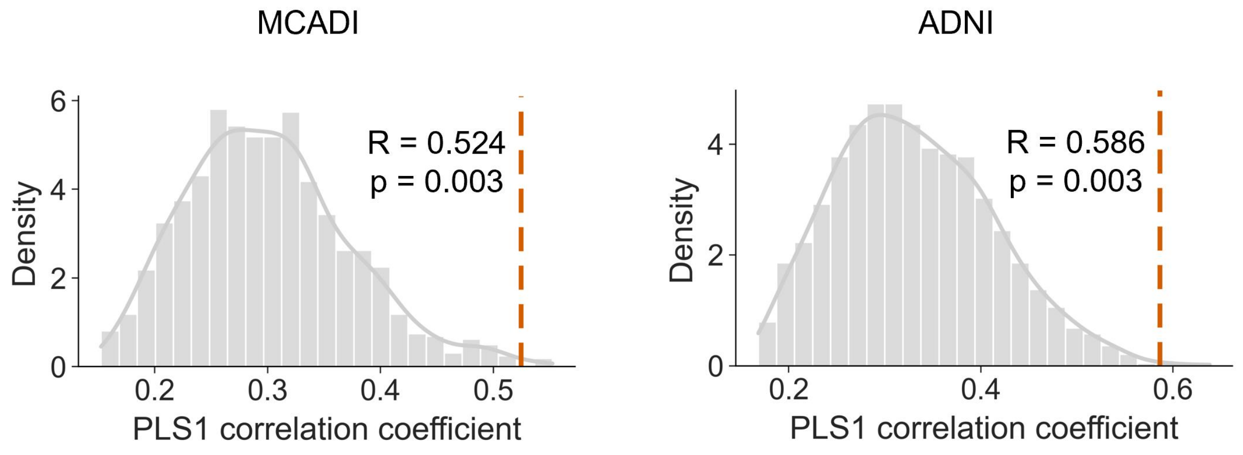

Figure S13. Spatial autocorrelation-preserving validation of imaging-transcriptomic associations. Null distributions generated using BrainSMASH-based spatial autocorrelation-preserving surrogate maps for imaging-transcriptomic association analyses in the MCADI and ADNI datasets.

Figure S14. Genetic underpinnings of MCI network heterogeneity in the ADNI dataset.

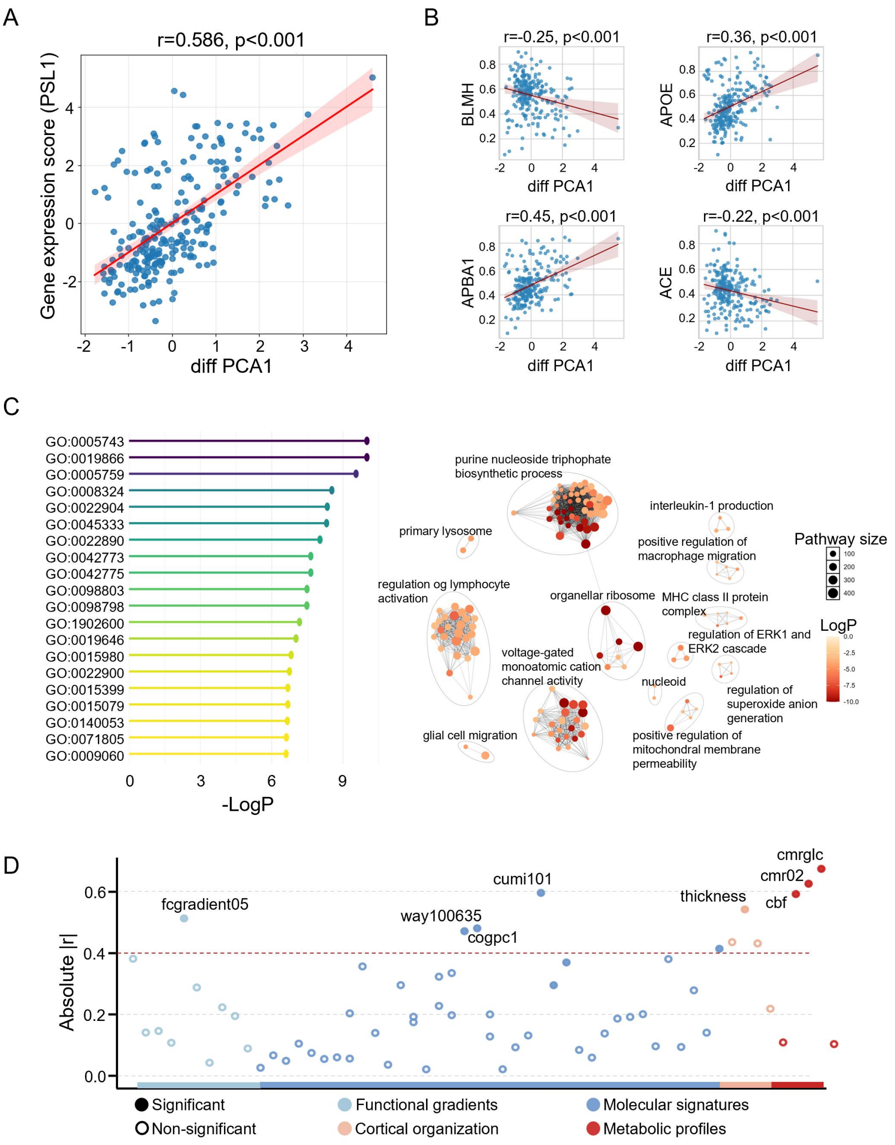

Figure S14. Genetic underpinnings of MCI network heterogeneity in the ADNI dataset. A) Correlation between the first principal component (PC1) of differential connections and regional gene expression derived from PLS regression. B) Significant correlations between PC1 loadings and regional expression of key AD risk genes. C) Top 20 significant GO terms and a pathway network of all FDR-significant terms, summarizing the functional roles of genes ranked by PC1-associated PLS weights. D) Spatial correlations between the PC1 genetic profile and multimodal brain maps.

Figure S15. Cross-dataset reproducibility of neuromaps association patterns.

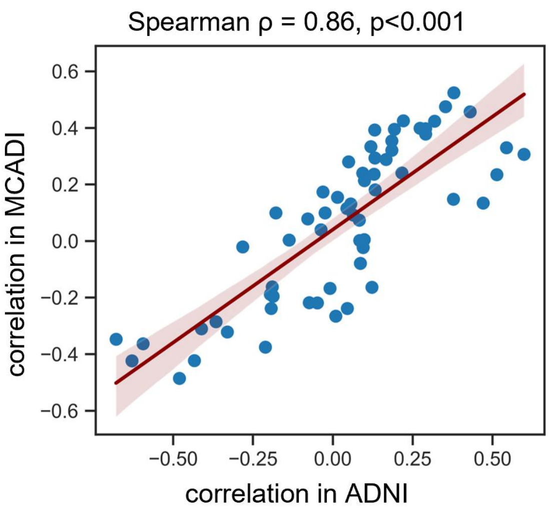

Figure S15. Cross-dataset reproducibility of neuromaps association patterns. Scatter plot showing the correspondence of PC1 correlation coefficients for neuromaps annotations between the ADNI and MCADI datasets.

References

1. Yesavage, J.A., Brink, T.L., Rose, T.L., Lum, O., Huang, V., Adey, M., and Leirer, V.O. (1982). Development and validation of a geriatric depression screening scale: a preliminary report. J Psychiatr Res *17*, 37-49. 10.1016/0022-3956(82)90033-4.

2. Morris, J.C. (1993). The Clinical Dementia Rating (CDR): current version and scoring rules. Neurology *43*, 2412-2414. 10.1212/wnl.43.11.2412-a.

3. Petersen, R.C., Smith, G.E., Waring, S.C., Ivnik, R.J., Tangalos, E.G., and Kokmen, E. (1999). Mild cognitive impairment: clinical characterization and outcome. Archives of neurology *56*, 303-308. 10.1001/archneur.56.3.303.

4. Feng, F., Wang, P., Zhao, K., Zhou, B., Yao, H., Meng, Q., Wang, L., Zhang, Z., Ding, Y., Wang, L., et al. (2018). Radiomic Features of Hippocampal Subregions in Alzheimer's Disease and Amnestic Mild Cognitive Impairment. Front Aging Neurosci *10*, 290. 10.3389/fnagi.2018.00290.

5. Zhou, B., Yao, H., Wang, P., Zhang, Z., Zhan, Y., Ma, J., Xu, K., Wang, L., An, N., Liu, Y., and Zhang, X. (2015). Aberrant Functional Connectivity Architecture in Alzheimer's Disease and Mild Cognitive Impairment: A Whole-Brain, Data-Driven Analysis. Biomed Res Int *2015*, 495375. 10.1155/2015/495375.

6. Wang, P., Zhou, B., Yao, H., Zhan, Y., Zhang, Z., Cui, Y., Xu, K., Ma, J., Wang, L., An, N., et al. (2015). Aberrant intra- and inter-network connectivity architectures in Alzheimer's disease and mild cognitive impairment. Sci Rep *5*, 14824. 10.1038/srep14824.

7. Zhang, Z., Liu, Y., Zhou, B., Zheng, J., Yao, H., An, N., Wang, P., Guo, Y., Dai, H., Wang, L., et al. (2014). Altered functional connectivity of the marginal division in Alzheimer's disease. Current Alzheimer research *11*, 145-155. 10.2174/1567205011666140110112608.

8. Yao, H., Zhou, B., Zhang, Z., Wang, P., Guo, Y., Shang, Y., Wang, L., Zhang, X., An, N., Liu, Y., and Alzheimer's Disease Neuroimaging, I. (2014). Longitudinal alteration of amygdalar functional connectivity in mild cognitive impairment subjects revealed by resting-state FMRI. Brain Connect *4*, 361-370. 10.1089/brain.2014.0223.

9. Guo, Y., Zhang, Z., Zhou, B., Wang, P., Yao, H., Yuan, M., An, N., Dai, H., Wang, L., Zhang, X., and Liu, Y. (2014). Grey-matter volume as a potential feature for the classification of Alzheimer's disease and mild cognitive impairment: an exploratory study. Neurosci Bull *30*, 477-489. 10.1007/s12264-013-1432-x.

10. Zhou, B., Liu, Y., Zhang, Z., An, N., Yao, H., Wang, P., Wang, L., Zhang, X., and Jiang, T. (2013). Impaired functional connectivity of the thalamus in Alzheimer's disease and mild cognitive impairment: a resting-state fMRI study. Current Alzheimer research *10*, 754-766. 10.2174/15672050113109990146.

11. Yao, H., Liu, Y., Zhou, B., Zhang, Z., An, N., Wang, P., Wang, L., Zhang, X., and Jiang, T. (2013). Decreased functional connectivity of the amygdala in Alzheimer's disease revealed by resting-state fMRI. European journal of radiology *82*, 1531-1538. 10.1016/j.ejrad.2013.03.019.

12. Wang, P., Zhang, X., Liu, Y., Liu, S., Zhou, B., Zhang, Z., Yao, H., Zhang, X., and Jiang, T. (2013). Perceptual and response interference in Alzheimer's disease and mild cognitive impairment. Clin Neurophysiol *124*, 2389-2396. 10.1016/j.clinph.2013.05.014.

13. Zhang, Z., Liu, Y., Jiang, T., Zhou, B., An, N., Dai, H., Wang, P., Niu, Y., Wang, L., and Zhang, X. (2012). Altered spontaneous activity in Alzheimer's disease and mild cognitive impairment revealed by Regional Homogeneity. NeuroImage *59*, 1429-1440. 10.1016/j.neuroimage.2011.08.049.

14. Yan, T., Wang, W., Yang, L., Chen, K., Chen, R., and Han, Y. (2018). Rich club disturbances of the human connectome from subjective cognitive decline to Alzheimer's disease. Theranostics *8*, 3237-3255. 10.7150/thno.23772.

15. Li, S., Yuan, X., Pu, F., Li, D., Fan, Y., Wu, L., Chao, W., Chen, N., He, Y., and Han, Y. (2014). Abnormal changes of multidimensional surface features using multivariate pattern classification in amnestic mild cognitive impairment patients. J Neurosci *34*, 10541-10553. 10.1523/JNEUROSCI.4356-13.2014.

16. Lu, J., Li, D., Li, F., Zhou, A., Wang, F., Zuo, X., Jia, X.F., Song, H., and Jia, J. (2011). Montreal cognitive assessment in detecting cognitive impairment in Chinese elderly individuals: a population-based study. J Geriatr Psychiatry Neurol *24*, 184-190. 10.1177/0891988711422528.

17. Guo, Q., Sun, Y., Yu, P., Hong, Z., and Lu, C. (2007). Norm of Auditory Verbal Learning Test in the Normal Aged in China Community. Chinese Journal of Clinical Psychology *15*, 132-134.

18. Hamilton, M. (1960). A rating scale for depression. Journal of neurology, neurosurgery, and psychiatry *23*, 56-62. 10.1136/jnnp.23.1.56.

19. Dozeman, E., van Schaik, D.J., van Marwijk, H.W., Stek, M.L., van der Horst, H.E., and Beekman, A.T. (2011). The center for epidemiological studies depression scale (CES-D) is an adequate screening instrument for depressive and anxiety disorders in a very old population

living in residential homes. Int J Geriatr Psychiatry *26*, 239-246. 10.1002/gps.2519.

20. Petersen, R.C. (2004). Mild cognitive impairment as a diagnostic entity. Journal of internal medicine *256*, 183-194. 10.1111/j.1365-2796.2004.01388.x.

21. McKhann, G., Drachman, D., Folstein, M., Katzman, R., Price, D., and Stadlan, E.M. (1984). Clinical diagnosis of Alzheimer's disease: report of the NINCDS-ADRDA Work Group under the auspices of Department of Health and Human Services Task Force on Alzheimer's Disease. Neurology *34*, 939-944. 10.1212/wnl.34.7.939.

22. McKhann, G.M., Knopman, D.S., Chertkow, H., Hyman, B.T., Jack, C.R., Jr., Kawas, C.H., Klunk, W.E., Koroshetz, W.J., Manly, J.J., Mayeux, R., et al. (2011). The diagnosis of dementia due to Alzheimer's disease: recommendations from the National Institute on Aging-Alzheimer's Association workgroups on diagnostic guidelines for Alzheimer's disease. Alzheimers Dement *7*, 263-269. 10.1016/j.jalz.2011.03.005.

23. Petersen, R.C., Doody, R., Kurz, A., Mohs, R.C., Morris, J.C., Rabins, P.V., Ritchie, K., Rossor, M., Thal, L., and Winblad, B. (2001). Current concepts in mild cognitive impairment. Archives of neurology *58*, 1985-1992. 10.1001/archneur.58.12.1985.

24. Choo, I.H., Lee, D.Y., Youn, J.C., Jhoo, J.H., Kim, K.W., Lee, D.S., Lee, J.S., and Woo, J.I. (2007). Topographic patterns of brain functional impairment progression according to clinical severity staging in 116 Alzheimer disease patients: FDG-PET study. Alzheimer Dis Assoc Disord *21*, 77-84. 10.1097/WAD.0b013e3180687418.

25. Li, J., Jin, D., Li, A., Liu, B., Song, C., Wang, P., Wang, D., Xu, K., Yang, H., Yao, H., et al. (2019). ASAF: altered spontaneous activity fingerprinting in Alzheimer’s disease based on

multisite fMRI. Science Bulletin. 10.1016/j.scib.2019.04.034.

26. Jin, D., Wang, P., Zalesky, A., Liu, B., Song, C., Wang, D., Xu, K., Yang, H., Zhang, Z., Yao, H., et al. (2020). Grab-AD: Generalizability and reproducibility of altered brain activity and diagnostic classification in Alzheimer's Disease. Hum Brain Mapp *41*, 3379-3391. 10.1002/hbm.25023.

27. Wu, Y., Zhang, Y., Liu, Y., Liu, J., Duan, Y., Wei, X., Zhuo, J., Li, K., Zhang, X., Yu, C., et al. (2016). Distinct Changes in Functional Connectivity in Posteromedial Cortex Subregions during the Progress of Alzheimer's Disease. Front Neuroanat *10*, 41. 10.3389/fnana.2016.00041.

28. Liu, J., Zhang, X., Yu, C., Duan, Y., Zhuo, J., Cui, Y., Liu, B., Li, K., Jiang, T., and Liu, Y. (2016). Impaired Parahippocampus Connectivity in Mild Cognitive Impairment and Alzheimer's Disease. J Alzheimers Dis *49*, 1051-1064. 10.3233/JAD-150727.

29. Liu, Y., Yu, C., Zhang, X., Liu, J., Duan, Y., Alexander-Bloch, A.F., Liu, B., Jiang, T., and Bullmore, E. (2014). Impaired long distance functional connectivity and weighted network architecture in Alzheimer's disease. Cereb Cortex *24*, 1422-1435. 10.1093/cercor/bhs410.

30. He, X., Qin, W., Liu, Y., Zhang, X., Duan, Y., Song, J., Li, K., Jiang, T., and Yu, C. (2014). Abnormal salience network in normal aging and in amnestic mild cognitive impairment and Alzheimer's disease. Hum Brain Mapp *35*, 3446-3464. 10.1002/hbm.22414.

31. Song, J., Qin, W., Liu, Y., Duan, Y., Liu, J., He, X., Li, K., Zhang, X., Jiang, T., and Yu, C. (2013). Aberrant functional organization within and between resting-state networks in AD. PLoS One *8*, e63727. 10.1371/journal.pone.0063727.
